# Predicting explorative motor learning using decision-making and motor noise

**DOI:** 10.1371/journal.pcbi.1005503

**Published:** 2017-04-24

**Authors:** Xiuli Chen, Kieran Mohr, Joseph M. Galea

**Affiliations:** School of Psychology, University of Birmingham, Birmingham, United Kingdom; Johns Hopkins University, UNITED STATES

## Abstract

A fundamental problem faced by humans is learning to select motor actions based on noisy sensory information and incomplete knowledge of the world. Recently, a number of authors have asked whether this type of motor learning problem might be very similar to a range of higher-level decision-making problems. If so, participant behaviour on a high-level decision-making task could be predictive of their performance during a motor learning task. To investigate this question, we studied performance during an explorative motor learning task and a decision-making task which had a similar underlying structure with the exception that it was not subject to motor (execution) noise. We also collected an independent measurement of each participant’s level of motor noise. Our analysis showed that explorative motor learning and decision-making could be modelled as the (approximately) optimal solution to a Partially Observable Markov Decision Process bounded by noisy neural information processing. The model was able to predict participant performance in motor learning by using parameters estimated from the decision-making task and the separate motor noise measurement. This suggests that explorative motor learning can be formalised as a sequential decision-making process that is adjusted for motor noise, and raises interesting questions regarding the neural origin of explorative motor learning.

## Author summary

Until recently, motor learning was viewed as an automatic process that was independent, and even in conflict with higher-level cognitive processes such as decision-making. However, it is now thought that decision-making forms an integral part of motor learning. To further examine the relationship between decision-making and motor learning, we asked whether explorative motor learning could be considered a decision-making task that was adjusted for motor noise. We studied human performance in an explorative motor learning task and a decision-making task which had a similar underlying structure with the exception that it was not subject to motor (execution) noise. In addition, we independently measured each participant’s level of motor noise. Crucially, with a computational model, we were able to predict participant explorative motor learning by using parameters estimated from the decision-making task and the separate motor noise task. This suggests that explorative motor learning can be formalised as a sequential decision-making process that is adjusted for motor noise, and reinforces the view that the mechanisms which control decision-making and motor behaviour are highly integrated.

## Introduction

Previously, human motor learning has mainly been examined through motor adaptation tasks in which participants are exposed to a novel perturbation during reaching movements [1–4]. The error reduction observed during these tasks has been conceptualised as a cerebellar-dependent supervised-learning process in which they learn through a sensory prediction error [3, 5, 6]. However, recent work has shown that motor learning is a far more complex process that can involve multiple mechanisms, including decision-making processes, taking place simultaneously [7–10].

One example of these motor-learning processes is reinforcement learning. This learning mechanism requires participants to explore their motor behaviour in order to identify actions that maximise expected future success/reward (in contrast with minimising the sensory prediction error). Despite being significantly slower and more variable than learning through a sensory prediction error, recent work has shown that participants are able to identify and adjust specific features of a movement, such as the curvature of a trajectory, simply through a reinforcement signal [8, 11–15]. Such explorative motor learning has been explained using reinforcement models in which learning is driven by a reward prediction error. This enables actions to be selected based on the probability of yielding future rewards [11, 13, 15, 16].

Arguably, it follows that explorative motor learning is simply a sequential decision task where the goal is to optimise reward in the face of task and sensory uncertainty. If so, participant behaviour on a matched high-level decision-making task should be predictive of performance during an explorative motor learning task. Previous work has compared high-level (economic) decision-making tasks with an equivalent motor lottery task [17, a review]. Some found that, in contrast to the well-documented sub-optimality in high-level (economic) decision-making [18], participants were able to perform near optimal decisions in a motor lottery task [19, 20]. For example, during simple pointing movements, participants hold an internal representation of motor noise uncertainty and compensate for this variability when planning a movement [19, 20]. However, others found that participants in a motor lottery task (where the uncertainty of outcomes were primarily due to motor noise) exhibited significant suboptimal choice patterns [17, 21]. Yet, the patterns of deviation from optimal choice were markedly different from those shown in high-level (economic) decision-making. Previous work highlights that one of the unique features that affect motor performance is a noisy motor system (motor noise). To our knowledge, most of these previous studies focused on binary or one-shot decision-making and its motor analogue. In contrast, here we ask if explorative motor learning is a sequential decision task that optimises reward in the face of task uncertainty, sensory uncertainty and motor noise uncertainty [15, 22]?

To explore this question, we investigated learning performance in an explorative motor learning task [13] and a decision-making task with a similar underlying structure with the exception that it was not subject to motor noise. We also took an independent measurement of each participant’s motor noise. We formulated the learning problem as a Partially Observable Markov Decision Process (POMDP) and built a computational model to solve the defined POMDP. The question we asked was whether we could predict participant explorative motor learning performance by fitting the model to the decision task performance and then adding each participant’s measured level of motor noise.

In addition, we were interested in whether we could predict motor learning performance as a function of gains and losses—one of the key concepts in the decision-making literature. In Prospect Theory [18], a theory of human decision-making, gains and losses are defined relative to a reference point that shifts with the decision context. For example [18], imagine a situation where a participant has just lost £2000 and is now facing a choice between a 100% chance of winning £1000 and a 50% chance of winning £2000 or nothing. If the participant’s reference frame had shifted to account for their recent loss, then they are likely to code the decision as choice between a 100% chance of losing £1000 and a 50% chance of losing £2000 or nothing. Understanding how people interpret gains and losses is important, because, for example, it has been shown that people are more adventurous in the latter representation (i.e., loss aversion, [18]). In the motor learning domain, research has shown that reward (positive feedback) and punishment (negative feedback) have multifaceted effects on motor learning [23]. Therefore, we were interested in understanding whether the ideas regarding gains and losses in decision-making were relevant to explorative motor learning.

## Results

### Behavioural analysis: Learning performance

**Fig 1:**
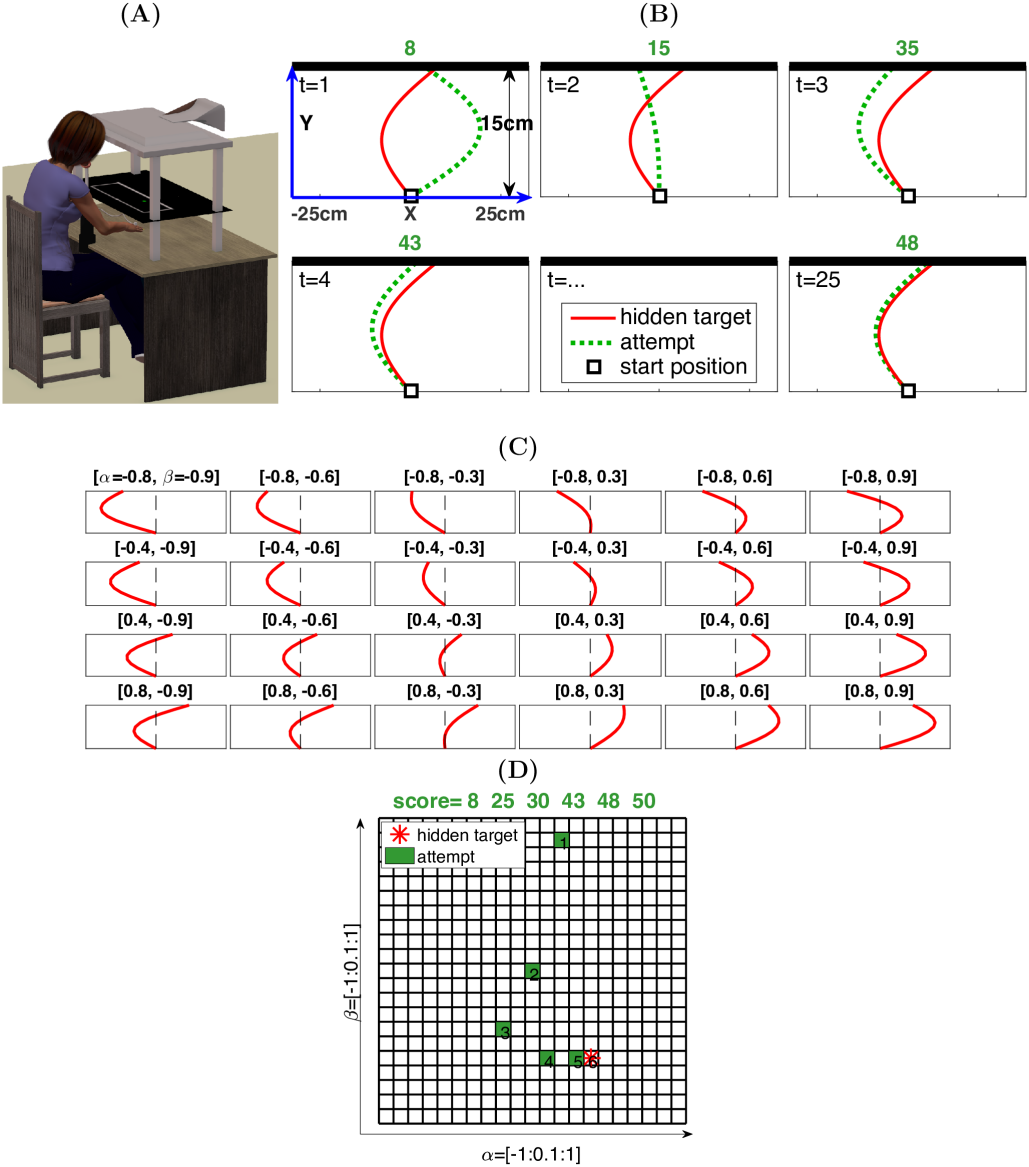
Experimental design for the motor learning (reaching) task and the decision-making task. **(A)** Illustration of the motor reaching task settings. **(B)** An example of the explorative motor learning in which a participant matched the reaching trajectory with a hidden target trajectory across 25 attempts. The red line represents the hidden target, while the green dashed lines represent the attempts. A score (points) was given after each attempt. **(C)** The 24 target trajectories used in the reaching task. The title of each panel includes the direction and curvature parameters for one target trajectory ([*α*, *β*]). These 24 targets were generated to be evenly distributed across the workspace. **(D)** Illustration of the decision-making task. Participants explored the cells (green) within the grid, defined by *α* and *β*, to find a hidden target cell (red asterisk). After each attempt (cell selection), a score (points) was provided.

We investigated performance during an explorative motor learning (reaching) task adopted from [13] and a novel decision-making (DM) task which had a similar underlying structure. In the reaching (MO) task (Fig 1A), participants were seated at a desk, looking down at a horizontal mirror that reflected task-related stimuli from a computer screen. The mirror blocked direct observation of the index finger, which was instead represented on the mirror via a circular green cursor. Participants were asked to draw trajectories by sliding their index finger from a central start position across the surface of the desk towards a target line (thick black line in Fig 1B) positioned in front of the start position. Participants made 25 attempts (green dashed lines in Fig 1B) to approximate each hidden target trajectory (red line). Each attempted trajectory resulted in a score that indicated the proximity of the attempted trajectory to the target trajectory. Both the target and the attempted trajectory were characterised by two parameters: direction and curvature (Fig 1C; Eq 1). The score for each attempt was calculated based on the errors between target and attempt in these two dimensions (Eq 2). The participants were instructed to adjust their movements’ direction and curvature based on the feedback to produce movements that were as close to the target trajectory as possible. Each participant attempted to match 24 different, invisible target trajectories that varied in both direction and curvature (Fig 1C).

We also designed a novel decision-making task. The objective was to capture the structure of the motor learning (reaching) task within a decision-making context that was uncontaminated by motor noise. The effect of motor noise on an aimed movement is that the outcome location is a probability density function centred on the goal [24]. In the decision-making task, participants interacted with an interface using a computer mouse. The interface consisted of a two-dimensional grid with cells (Fig 1D). The horizontal and vertical dimension reflected two parameters: *α* and *β* respectively, akin to the direction and curvature parameters in the reaching task. The parameter values were assigned to the cells in a spatially ordered manner. Each cell of the grid therefore corresponded to a unique combination of the two parameters. When one of the cells (i.e., one parameter pair) was chosen as a target cell, the score associated with each of the cells was then calculated using the same score function (Eq 2) as in the reaching task. Once a cell was chosen (mouse-clicked), an associated score would appear in the feedback window at the top of the screen. Similar to the reaching task, participants were required to explore different cells (parameter pairs) based on the feedback to find the cell that was as close to the target cell as possible. Participants were asked to search for a series of 24 hidden target cells.

In both tasks, the 24 target trajectories/cells were randomly divided into two feedback conditions (12 of each): a positive feedback condition and a negative feedback condition. In the positive feedback condition, points ranged from 0 to 50 (Eq 2), with greater magnitude indicating greater similarity between the attempted and target trajectory (50 for the target). In the negative feedback condition, points ranged from -50 to 0 (Eq 2), with greater magnitude indicating reduced similarity between the attempted and target trajectory (0 for the target). Hence, the goal for the positive feedback condition was to achieve 50 points, whereas for the negative feedback condition it was to achieve 0 points (i.e., avoiding losing points). Participants were told which of the two feedback conditions they were in at the beginning of each target search.

**Fig 2:**
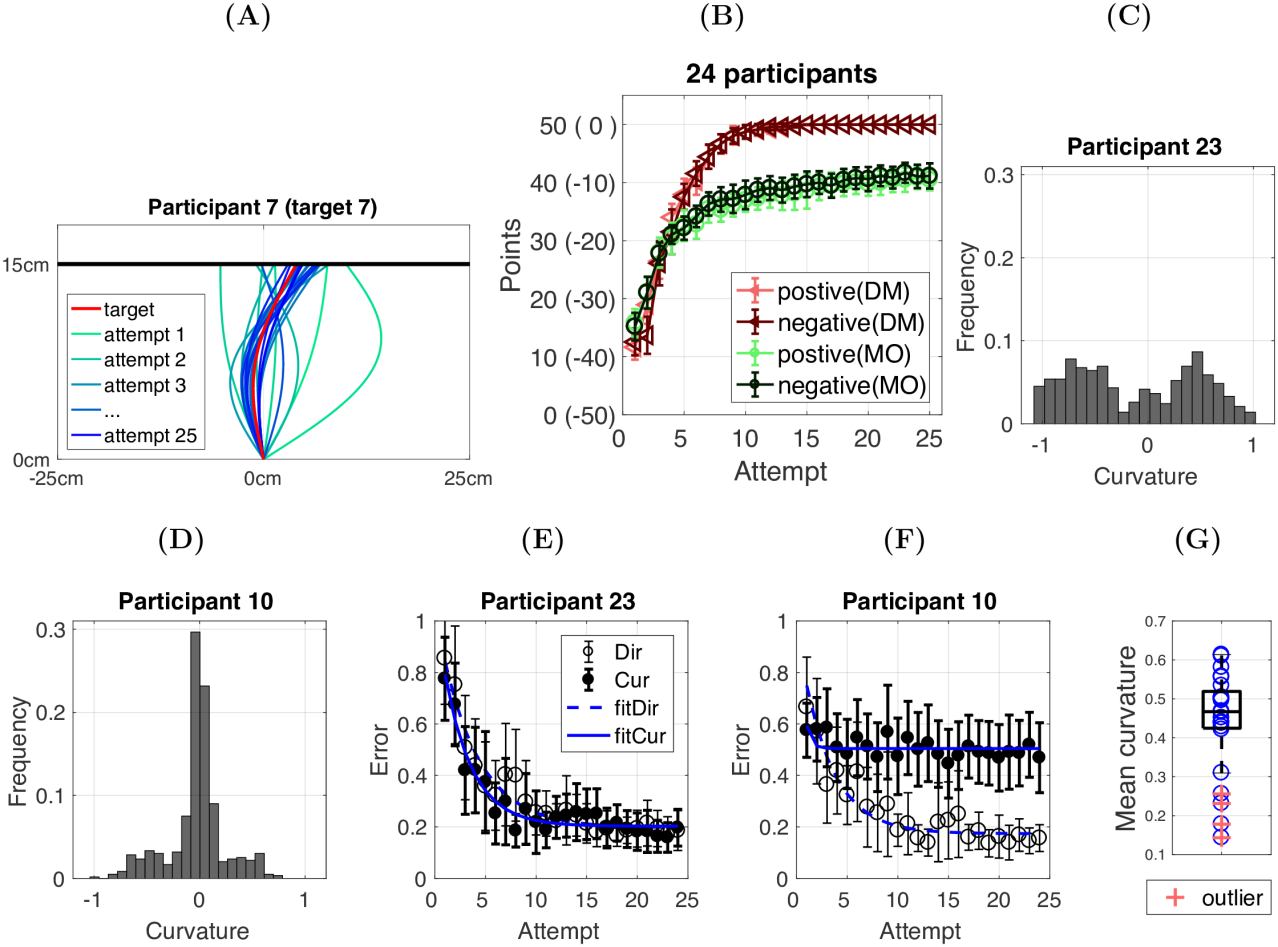
Behavioural learning performance for the decision-making and the reaching task. **(A)** Representative participant data showing how a reaching trajectory is gradually updated to match the hidden target trajectory (red). The colours of the lines indicate the sequence of attempts (ranging from green to blue), with later attempts being closer to the target trajectory. **(B)** Learning curves (positive and negative feedback conditions) for the participants in the decision-making task (DM) and the motor learning/reaching task (MO). Points achieved (y-axis) are plotted against the number of attempts (1-25). The dark red-triangle line and dark green-circle line represent the negative conditions in the DM and MO task respectively, while the light red-triangle line and light green-circle line represent the positive conditions. Error bars indicate 95% confidence intervals (CI) across 24 participants. **(C-D)** Two representative participants in terms of their curvature exploration. The curvature parameter (x-axis) ranges from −1 to 1, where −1 = ‘curve to the left’, 1 = ‘curve to the right’, and 0 = ‘straight movement’. The participant in (C) evenly explored the curvature dimension, while the participant in (D) concentrated on straight movements with little curvature. **(E-F)** Two representative participants in terms of their error reduction in both the direction (open circle) and curvature (solid circle) dimensions plotted against the number of attempts. For the participant in (E), the error in both dimensions was reduced to a relatively low level, while for the participant in (F) the error in curvature remained high. The latter was due to the lack of exploration in the curvature dimension as shown in panel (D). **(G)** Each participant’s mean curvature across all movements during the reaching task (blue circles; the absolute values were used for the movements with negative curvature). Four participants (10,16,18,22) were identified as outliers (red crosses). The blue circles (mean curvature values) were counted as outliers if they were larger than *q*3 + 0.15(*q*3 − *q*1) or smaller than *q*1 − 0.15(*q*3 − *q*1), where *q*1 and *q*3 were the 25*^th^* and 75*^th^* percentiles respectively.

Analysis of the points achieved, across both tasks, showed that participants were able to update their behaviour, based on the feedback, and produce actions that were close to the target trajectory/cell (Fig 2A and 2B). First we examined whether participant performance was different between the positive and negative feedback conditions within both tasks. To do so, we averaged each participant’s performance across all target trajectories/cells that were experienced with either positive or negative feedback (Fig 2B). We fitted the exponential function, *y* = *ae*^−*bx*^ + *c*, to each participant’s average learning curve in each condition (across 12 targets) (Decision-Making: *R*^2^ = 0.97 ± 0.02; reaching: *R*^2^ = 0.89 ± 0.10). Paired t-tests on the three parameters (a, b, c) revealed no significant differences between positive and negative feedback conditions in either the decision-making or reaching task (Table 1).

**Table 1:** Comparison of learning performance between the positive and negative feedback conditions. Paired t-test results on the three parameters (a,b and c in *y* = *ae*^−*bx*^ + *c*) between the positive and negative feedback conditions within each of the tasks.

	a (Positive vs Negative)	b (Positive vs Negative)	c (Positive vs Negative)
Decision-Making	**P**: M = -46.86; SD = 4.02 **N**: M = -47.22; SD = 2.75 t(23) = 0.36; p = 0.72; d = 0.07	**P**: M = 0.23; SD = 0.07 **N**: M = 0.23; SD = 0.06 t(23) = -0.26; p = 0.80; d= -0.05	**P**: M = 50.62; SD = 1.61 **N**: M = 50.18; SD = 1.47 t(23) = 1.11; p = 0.28; d = 0.23
Reaching	**P**: M = -29.37; SD = 8.10 **N**: M = -30.89; SD = 9.79 t(23) = 0.60; p = 0.55; d = 0.12	**P**: M = 0.30; SD = 0.18 **N**: M = 0.24; SD = 0.11 t(23) = 1.06; p = 0.30; d = 0.21	**P**: M = 39.44; SD = 5.58 **N**: M = 40.96; SD = 7.13 t(23) = -0.84; p = 0.41; d = -0.17

Further analysis regarding the effect of positive and negative feedback is provided at the end of the results section. However, for the following analysis, we pooled data from the positive and negative feedback conditions by simply defining a negative score as its positive equivalent. For example, a score of -40 (10 points above the minimum point -50) in the negative condition was equivalent to 10 (10 points above the minimum point 0) in the positive condition (Fig 2B). Therefore, we then had one average learning curve (across 24 targets) for each participant in each of the tasks. Next we compared the learning performance across tasks (Fig 2). In the decision-making task, starting from 12.08 ± 6.05, the average points achieved for each target was 49.98 ± 0.31. For the reaching task, starting from 15.92 ± 4.42, the average points achieved for each target was 40.96 ± 4.67. Although participants began with a similar score across tasks, they achieved significantly more points in the decision-making task (*t*(23) = 9.49, *p* < 0.001, *d* = 2.74).

We also noticed that some of the participants failed to explore the curvature dimension in the reaching task. Specifically, a small subset of participants produced straight movements with little curvature (Fig 2D). This resulted in significantly greater error remaining in the curvature dimension (Fig 2F), and thus substantially lower points being achieved. Having quantified the amount of curvature explored during the reaching task, 4 out of the 24 participants (10, 16, 18, 22) could be considered as outliers (Fig 2G). For the following analysis, we removed these 4 participants unless stated otherwise.

### Behavioural analysis: Action change and error reduction

**Fig 3:**
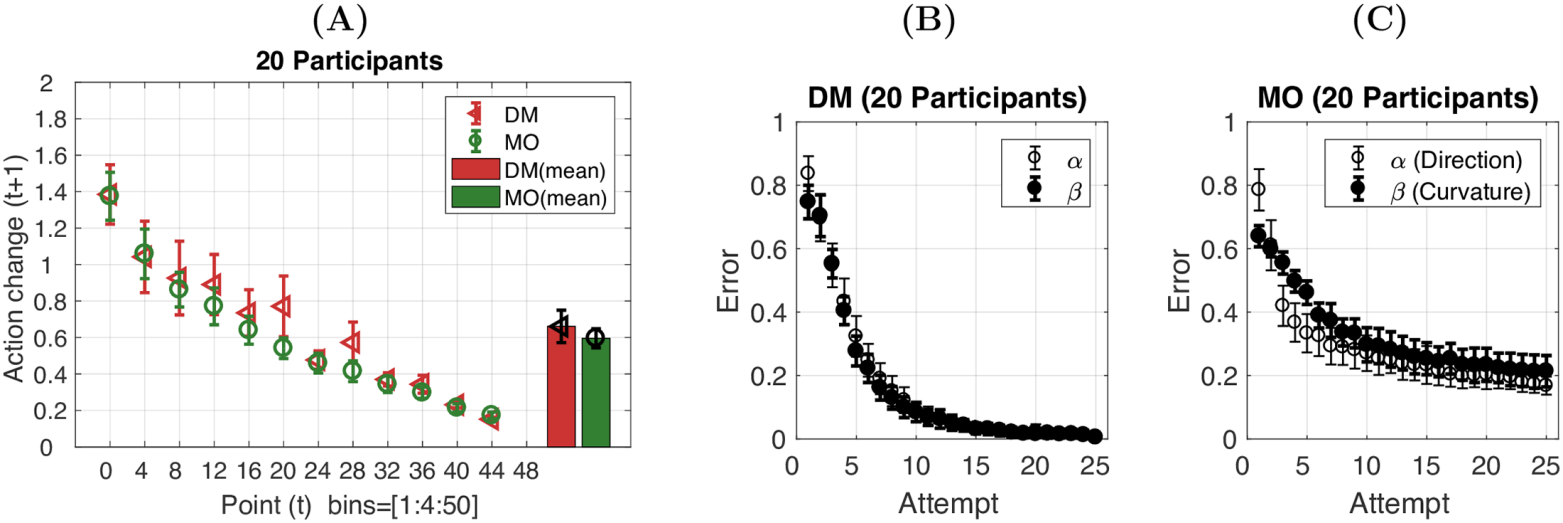
The score-effect on action selection and error reduction in the decision-making task (DM) and the reaching task (MO). **(A)** Action change on attempt t+1 (y-axis) following a score (points) received on attempt t (x-axis) in the DM task (red) and MO task (green). Bar plot represents average across points. **(B-C)** Error in *α* and *β* (y-axis) plotted against the number of attempts (x-axis) in the DM task (B) and MO task (C). Error bars in all panels represent 95%CI across 20 participants.

The aim of both tasks was to find the target by exploring a range of actions. As the proximity of an action to the target was indicated by the number of points, the exploration process may have been performed by avoiding the actions with bad outcomes (low reward, high punishment) and reinforcing the actions with good outcomes (high reward, low punishment). Thus we expected to see participants make larger action changes after receiving lower points and smaller action changes after receiving higher points. Using the *α* and *β* parameters from each action, we determined action change, ∇*A*, between two successive actions: *a*_*t*_ = [*α*_*t*_, *β*_*t*_] and *a*_*t*+1_ = [*α*_*t*+1_, *β*_*t*+1_] as Euclidean distance between two points, i.e., $\nabla A=|\sqrt{{\left(\alpha_{t}-\alpha_{t+1}\right)}^{2}+{\left(\beta_{t}-\beta_{t+1}\right)}^{2}}|$. As shown in Fig 3A, the action change decreased as a function of score in both tasks. Interestingly, although the actions were in different forms across the tasks, the amount of action change (in terms of the Euclidean distance measurement) given the levels of score was quantitatively similar across the tasks. Paired t-tests revealed no significant difference in the average action changes between the DM and MO tasks (t(19) = 1.33, p = 0.20, d = 0.42; Bars in Fig 3A).

One pressing question is how the action change looked in terms of *α* and *β* within and across tasks. To examine this, we first fitted the exponential function, *y* = *ae*^−*bx*^ + *c*, to each participant’s error reduction learning curves (examples shown in Fig 2E and 2F; DM: *R*^2^ = 0.96 ± 0.02 [*α*], *R*^2^ = 0.92 ± 0.20 [*β*]; MO: *R*^2^ = 0.84 ± 0.20 [*α*], *R*^2^ = 0.80 ± 0.26 [*β*]). Secondly, three two-way (IV1:task = DM vs MO; IV2:dimension =*α* vs *β*) repeated measures ANOVA were performed for the parameters a, b and c respectively. The results showed that the error reduction rate (b) and plateau (c) were not significantly different across *α* and *β* within each of the tasks (S1 Table). In both tasks, the errors in both dimensions were equally weighted to determine the feedback score (Eq 2). Hence, the participants learnt to treat these two dimensions equally in order to achieve maximal points. On the other hand, the error reduction rate (b) and plateau (c) were different across the tasks (S1 Table). We postulate that this difference was primarily due to the fact that the reaching task required participants to overcome uncertainty involved in the execution of the planned trajectories (motor noise) and the lack of visual information of the executed action that was associated with the feedback score.

### Behavioural analysis: Motor noise measurement

**Fig 4:**
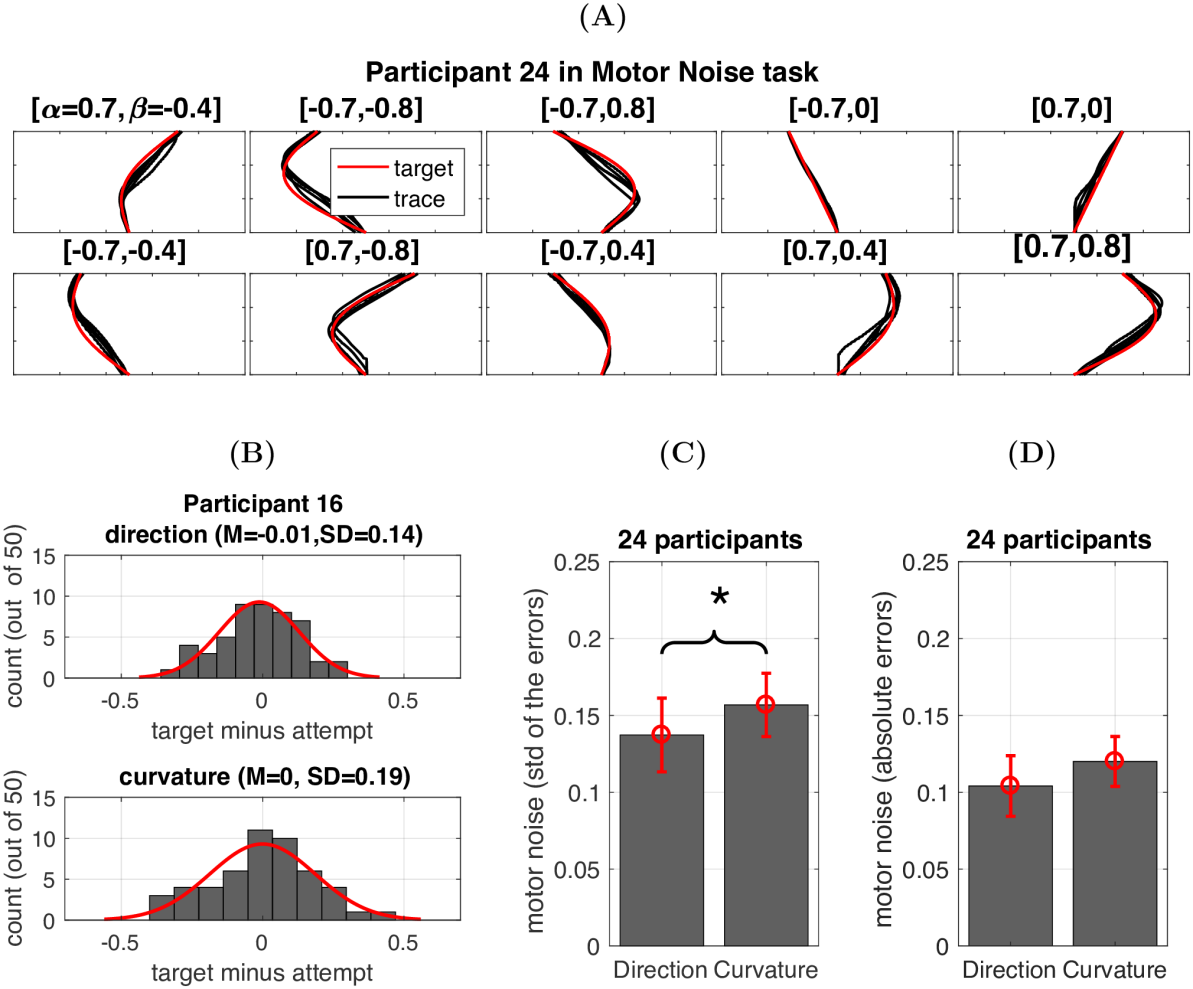
Motor noise measurement task. **(A)** Data for one representative participant during the motor noise measurement task. The red lines indicate the target trajectories that the participants were asked to trace within a certain time window. The black lines in each panel represent the 5 attempts made by one participant. **(B)** Histograms of the errors in direction and curvature for one representative participant. **(C)** Motor noise in direction and curvature across 24 participants. *p = 0.049. **(D)** The absolute error in the direction and curvature dimensions due to motor noise.

To examine the role of motor noise in the explorative motor learning task, we obtained a measure of motor noise for each participant. In the motor noise measurement task, unlike the main motor learning task where the target trajectories were hidden, a series of trajectories was displayed on the screen (red lines in Fig 4A). For each displayed trajectory, the participants were asked to trace it within a specific time window (> 700ms and < 1500ms). Five traces were performed for each trajectory (black lines in Fig 4A).

By comparing the direction and curvature parameters of each trace with the target parameters, we obtained one direction error and one curvature error for each trace. Therefore, we had 5 pairs of errors for each target trajectory (5 traces). Each participant was asked to trace 10 target trajectories. Hence, we collected 50 errors in the direction and 50 errors in the curvature (Fig 4B). For each participant, we calculated the standard deviation across the errors in the direction and curvature parameters and used these two standard deviations as our measure of their motor noise in the direction and curvature dimensions, respectively (Fig 4B). As shown in Fig 4D, although participants were encouraged to replicate the trajectories displayed on the screen, the average errors made in both dimensions were significantly larger than zero (Dir: 0.10 ± 0.05; Cur: 0.12 ± 0.04).

### Behavioural analysis: Exploration and motor noise

**Fig 5:**
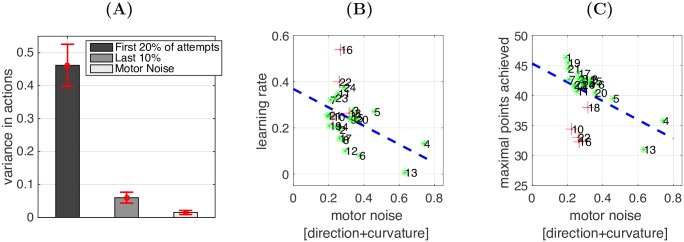
Exploration and motor noise. **(A)** How movement variance in the movements changes during the course of the learning process, compared with the variance observed in the motor noise task. **(B)** Learning rate and **(C)** maximal points achieved plotted against the variance observed in the motor noise task across participants (x-axis). Each dot represents one participant, indexed with the participant ID; Red crosses in (C) are the participants who failed to explore the curvature dimension (concentrated on straight movements with little curvature) and were identified as outliers. The least-squares line (blue dash line) is with the outliers removed.

Next, we examined how each participant’s level of motor noise correlated with their ability to ‘find’ the hidden target trajectory. First, the measure of variance from the motor noise task provided an estimate of how accurate a participant could replicate a planned movement trajectory. During the reaching task, movement variance was initially relatively high as participants explored the space of possible trajectories (including both the exploration variance and motor noise variance) (Fig 5A). However, by the end of each target search movement variance had decreased toward to a level observed in the motor noise task (although still higher than the variance purely due to motor noise). More importantly, we found that the level of variance observed in the motor noise task was negatively associated with motor learning performance across participants. Specifically, we fitted an exponential function, *y* = *ae*^−*bx*^ + *c*, to each participant’s average learning curve across all the targets in the reaching task (*R*^2^ = 0.94 ± 0.05). A Pearson correlation indicated that there was a negative correlation between motor noise and the learning rate parameter *b* (r = -0.47, n = 20, p = 0.022; Fig 5B), and maximal points achieved (r = -0.49, n = 20, *p* = 0.015; Fig 5C).

### Model analysis

**Fig 6:**
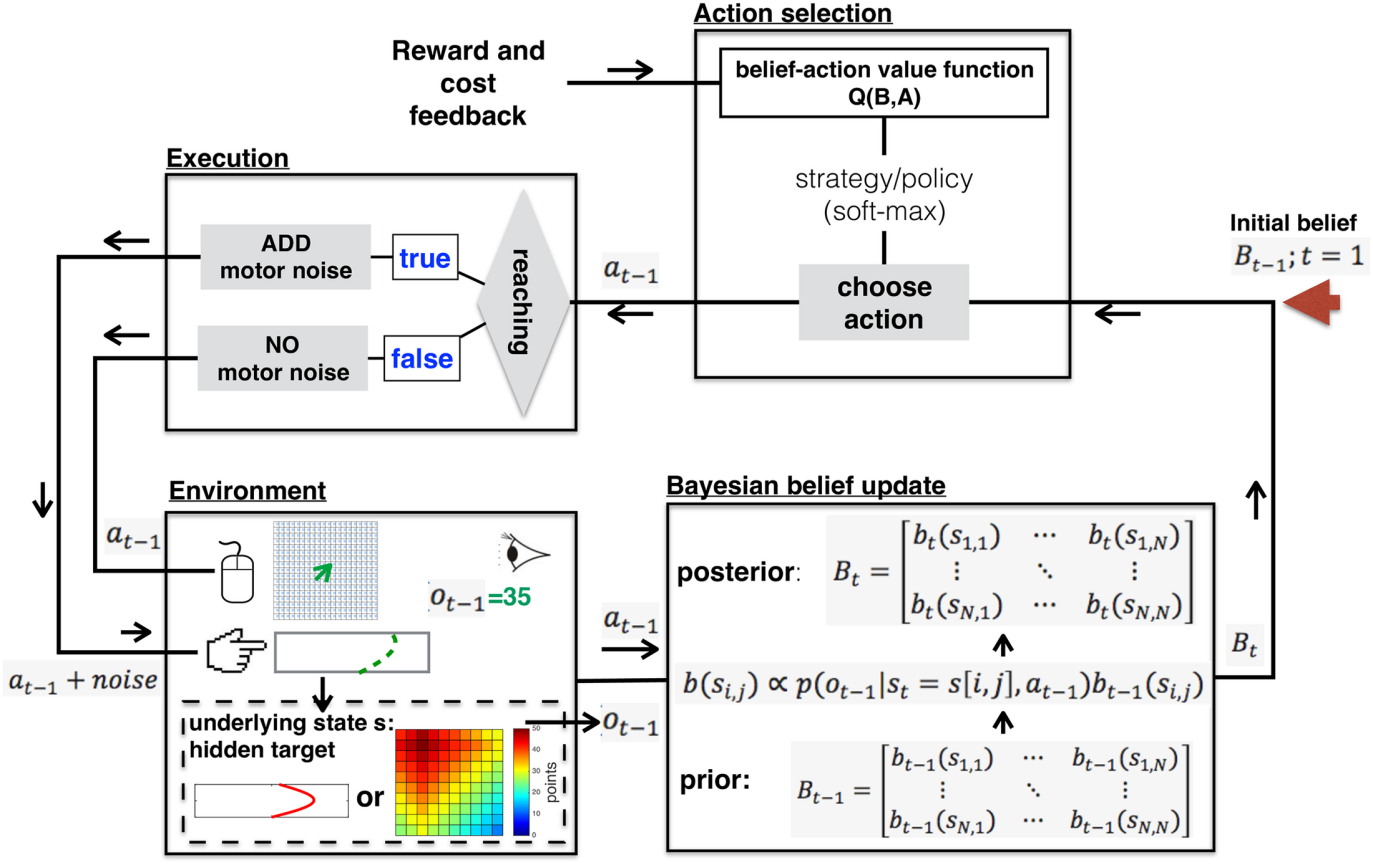
An illustration of the model for the decision-making task and the explorative motor learning task. On each trial, a hidden target is chosen (Environment). That is, the environment is in a state, which is not directly observable. The model starts with an initial uniformly distributed belief state (illustrated with the red arrow on the top right). On each time step, given an belief, the model then chooses an action based on the belief-action value function (Action selection). Subsequently, the action is executed (Execution). Decision-making task actions are performed without motor noise; the model is able to choose the selected action accurately. Reaching actions are performed with motor noise; there is uncertainty between the selected and executed action. Once the action is executed, the environment gives observable feedback (*o*_*t*−1_ = 35 in the figure). The action and observation are then used to update the belief (Bayesian belief update). The update is constrained by the fact that participants were naïve to the score function used. We modelled this uncertainty using the likelihood uncertainty parameter (Γ; Eq 3). A new cycle then starts with the new belief state (Bt).

The main purpose of this study was to test whether explorative motor learning and decision-making could both be understood as a sequential decision process that optimises reward given task, sensory and/or motor uncertainty. To this end, we framed the learning problem as a Partially Observable Markov Decision Process (POMDP) [25] and built a computational model to solve (approximately) the defined POMDP. The POMDP framework has been proposed to model a variety of real-world sequential decision problems [25–30], and provides a general mathematical framework that captures the interaction between an agent and a stochastic environment (Fig 6). It suggests an interpretation of participant behaviour in terms of maximising total expected future reward.

An informal description of the decision-making task as a POMDP is given in what follows (for a formal description see the Methods). There is a set of states, each of which corresponds to an event in which the target is one of the cells in the grid (Fig 1D). As in the experiment, the task is divided into episodes; each episode consists of 25 time steps (attempts) to find a hidden target cell. On each episode, one of the cells is randomly chosen as the hidden target cell. That is, the environment is in one of the states (Environment; Fig 6); and the state is not directly observable. On each time step within one episode, the model chooses an action (i.e., which cell to click) based on a control strategy so as to maximise the expected future reward (Action selection; Fig 6). After taking an action, the model receives two signals from the environment: an observation and a reward (cost if the value is negative). In our case, the observation and reward are equal, which is the feedback score (points).

**Fig 7:**
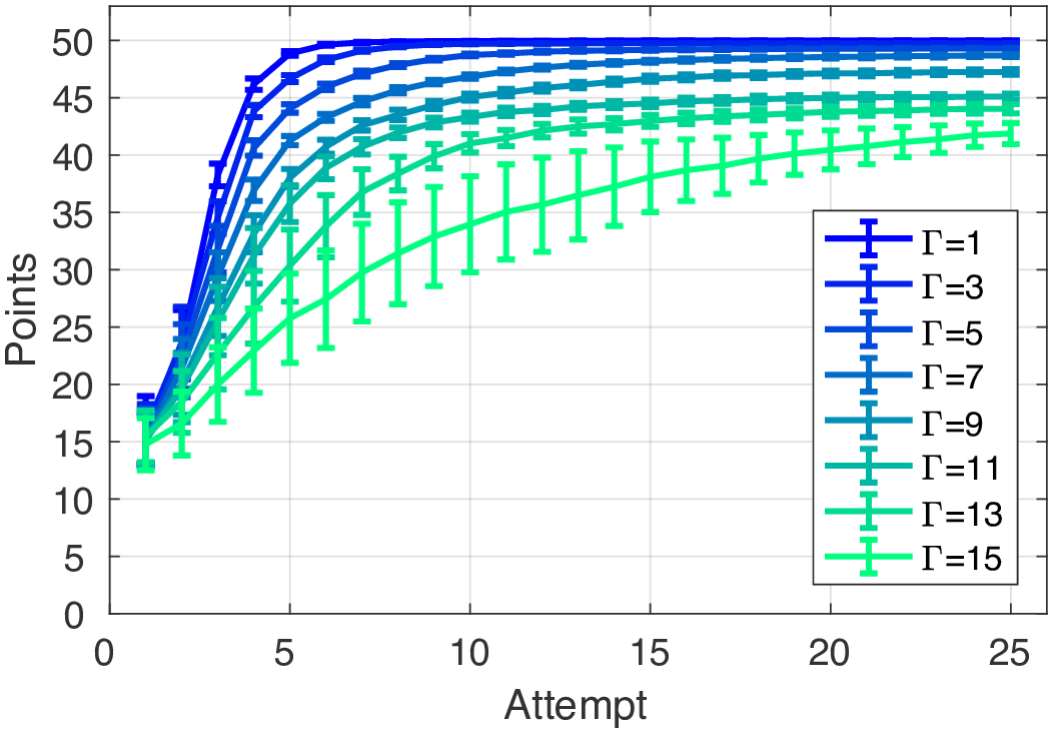
The effects of likelihood uncertainty parameter (Γ) on the speed at which the target is found. The models, with a range of likelihood uncertainty values (Γ: 1-15), were given the same set of the target cells as the participants. Model predictions show that an increasing amount of likelihood uncertainty caused learning (ability to locate the hidden target and achieve 50 points) to be slower and often incomplete after 25 attempts. Error bars represent model performance variance (95% CI) across the 24 targets. The model’s performance for each target was averaged over 100 runs.

Given the defined POMDP, an algorithm can then be used to acquire the optimal control strategy for action selection. In our model, an approximated optimal control strategy was acquired (more details in Methods). Framing the model as a POMDP allows for the calculation of the optimal policy given the theoretical constraints [31]. Constraints include the uncertainty in the sensory input and the uncertain effect of executing an action. The behaviour predicted by the optimal policy is therefore the rational behaviour given the constraints. The POMDP framing thereby serves the goal of drawing a causal relationship between the theoretical constraints and the behaviour (assuming rationality, [32]). For the decision-making task, we assumed that participant performance was constrained by the fact they were naïve to the underlying equation used to generate the score. In other words, participants were unsure how the current score (received by selecting a certain cell) related to the position of the target cell. This uncertainty was represented in our model by a likelihood uncertainty parameter (Γ; Eq 3). Crucially, this was the only free model parameter for the decision-making task. Initially, we ran the model and examined the effect of increasing likelihood uncertainty on learning rate. As shown in Fig 7, a model with a likelihood uncertainty of 1 would find the target after approximately 7 attempts, with increasing uncertainty causing a gradual decline in the speed at which the target was found.

#### Model performance: Learning curve

**Fig 8:**
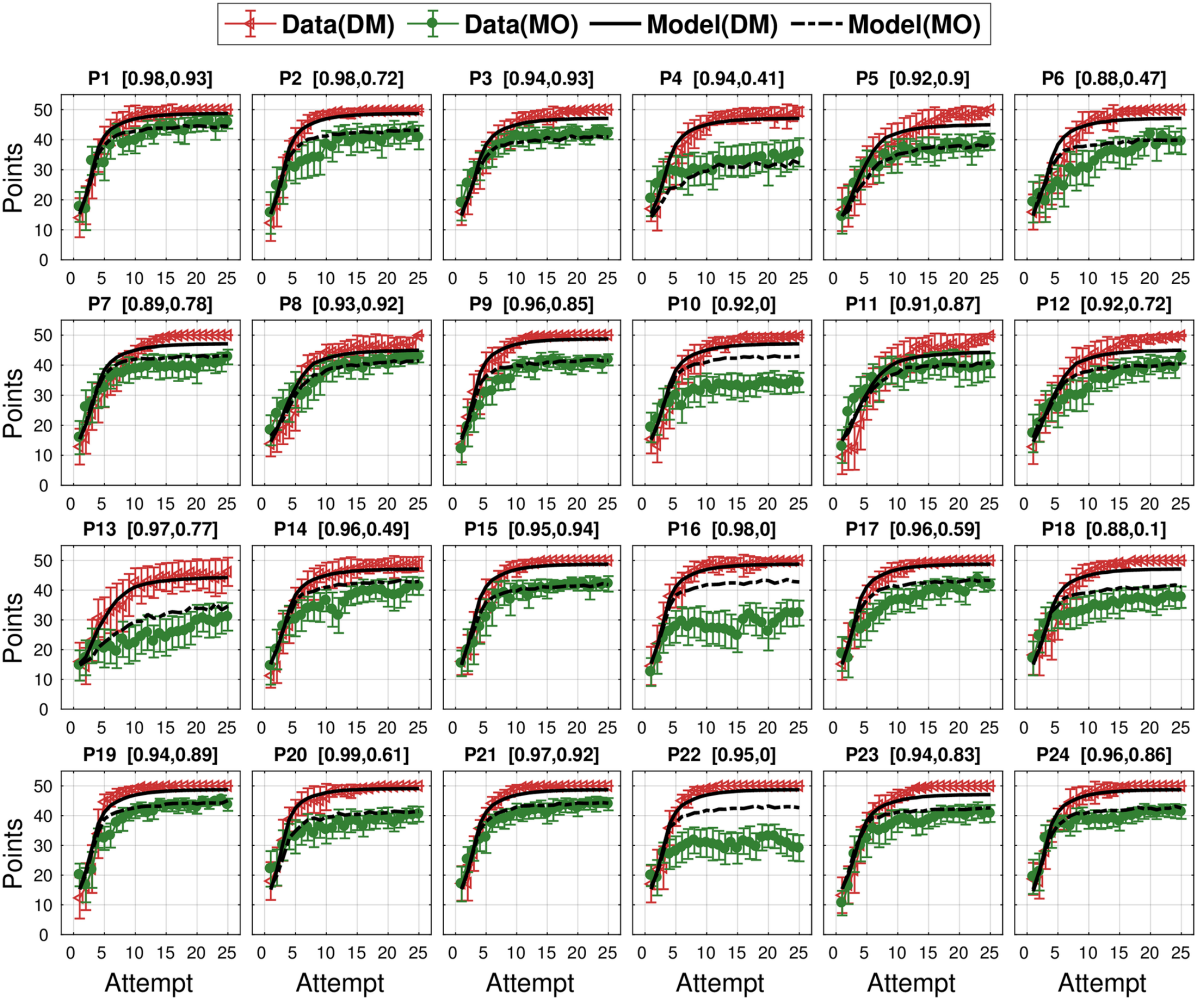
Model predictions for each participant’s learning curves in the DM and MO tasks. Each panel represents a single participant. Red lines represent the DM data. Green lines represent the MO data. The black and black dashed lines are model predictions for the DM task and the MO task respectively. Each title includes *R*^2^ for the DM task between the model and data (left), and *R*^2^ for the MO task between the model prediction and human data (right). The error bars represent 95% CI across 24 targets.

The best-fitting likelihood uncertainty parameter was found for each participant based on their individual learning curve averaged over 24 targets in the DM task. Specifically, we found the likelihood uncertainty parameter Γ which produced a learning curve that best-fit (maximum *R*^2^) each participant’s average learning curve (Fig 7). The search range was from 1 to 15; none of the best fits had values at the extreme of this range. Fig 8 shows that it was possible to fit all 24 participants learning behaviour in the DM task. Across 24 participants the average *R*^2^ between the model’s fitted decision-making learning curves and the participants’ actual data was 0.95 ± 0.03. Across participants, the best-fitted likelihood uncertainty value was 8.63 ± 2.46 (S3 and S4 Figs provide an example of a representative participant’s attempt-by-attempt performance to each target along with the model’s prediction).

We then built a model to predict each participant’s performance in the MO task. The model used each individual’s likelihood uncertainty (estimated from their DM task performance), and each individual’s noise parameters for direction and curvature (estimated from the motor noise measurement task). Therefore, this model was not fitted to the MO data but predicted it based on parameters derived from the decision-making and motor noise tasks. This predictive model was able to explain 0.63 ± 0.34 of the variance across the 24 participants (Fig 8). As mentioned, 4 of the participants were considered to be outliers. Having removed these 4 outliers, the model was able to explain 0.76 ± 0.19 of the remaining 20 participants’ variance within the MO task.

#### Model performance: Action change and error reduction

**Fig 9:**
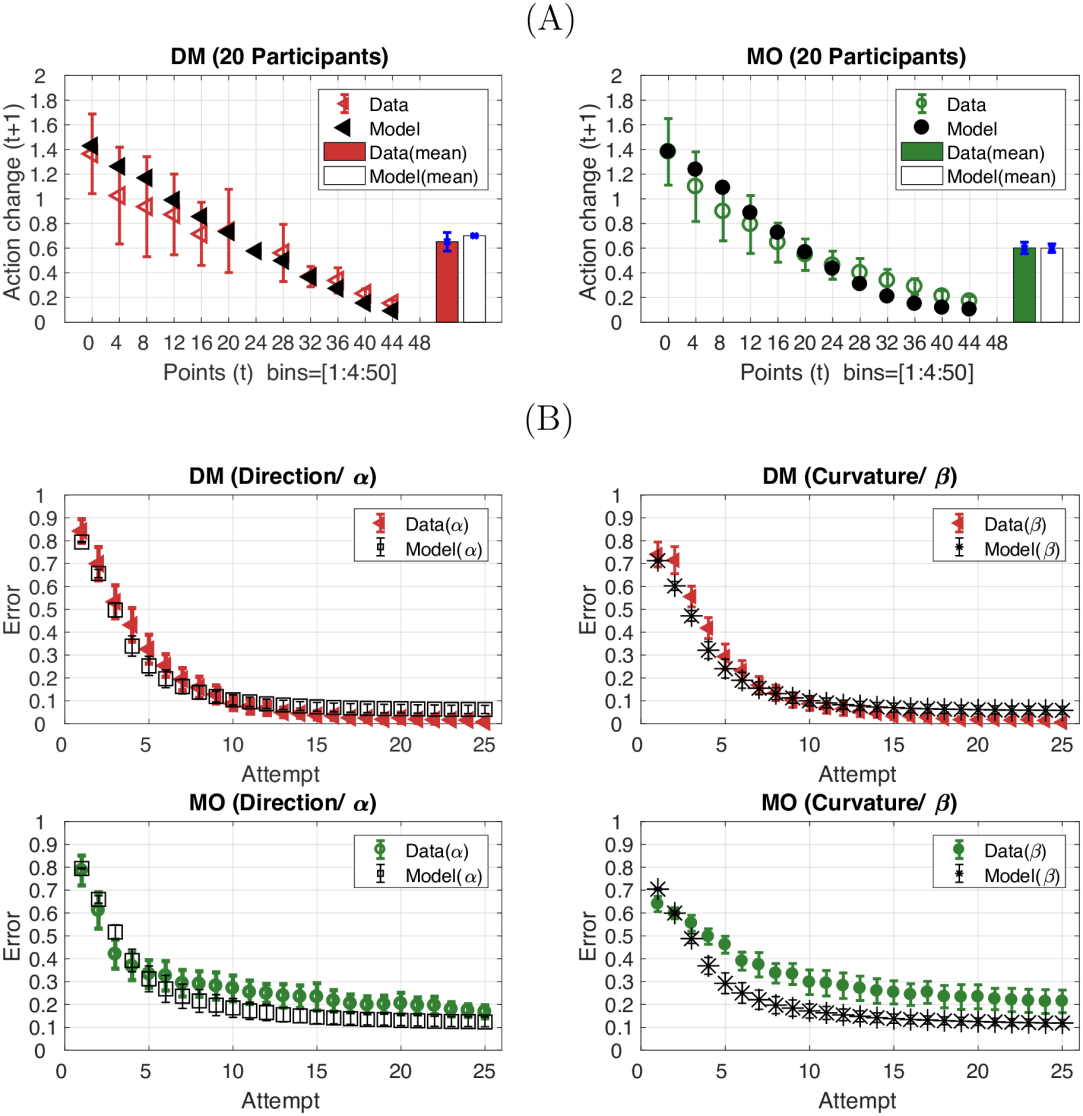
Model performance: The score-effect on action selection and error reduction in the DM and MO tasks. **(A)** Action change on attempt *t* + 1 (related to the action at step *t*, y-axis) following a score (points) received on attempt *t* (x-axis) in the DM (red) task and the MO task (green). The model predictions are also provided (black). **(B)** Error in *α* or *β* (y-axis) plotted against the number of attempts (x-axis) in the DM task and the MO task. Error bars in all panels represent 95%CI across 20 participants.

Next we examined model performance in terms of predicting the score-effect on action selection and error reduction across attempts. Similar to participant performance, the model predicted action change to decrease as a function of score in both the DM task (*R*^2^ = 0.88, Fig 9A left) and the MO task (*R*^2^ = 0.92, Fig 9A right). The model was also able to predict the error reduction observed across attempts in both dimensions and tasks (Fig 9B, DM: *α*: *R*^2^ = 0.95, *β*: *R*^2^ = 0.93, MO: *α*: *R*^2^ = 0.83, *β*: *R*^2^ = 0.39).

#### Model performance: Predictions for each target

We noticed that participant performance was systematically different across the different targets. For the DM task, we noticed that differences in target cell location appeared to be associated with differences in starting position and learning rate (S1 Fig). However, plateau performance was similar across target cell locations, perhaps owing to the fact that most participants found most targets within 25 attempts. We examined whether the model captured the variance in performance across the targets. S1 Fig shows the model’s predicted learning curves against participants’ for all 24 target cells and highlights that the model was able to capture the variance across targets (*R*^2^ = 0.903 ± 0.05). For the reaching task, participant performance also varied depending on the shape of the target trajectory. S2 Fig shows that performance varied in most dimensions, including start points, plateaus and the learning rate. The model was able to explain 0.57 ± 0.3 of the variance across targets. Interestingly, the model predicted faster learning rates and higher plateaus than what was achieved by the participants for a number of trajectories. These trajectories appear harder for participants than for the model. These trajectories had higher curvature than other trajectories and also began on one side of the central line and finished on the opposite side (e.g., trajectory 6,12,13,19 in S2 Fig). This may indicate that the theoretical assumptions of the model are under constrained.

#### Model performance: Alternative models for the reaching task

In order to understand the benefits of modelling individual motor noise and individual Gamma (Γ, estimated from the DM task) for predicting participant performance in the MO task, we compared our main model (i.e., a model with individual Gamma and individual motor noise) with two alternatives: a model with individual Gamma and average group motor noise (alternative Model 1) and a model with average group Gamma and individual motor noise (alternative Model 2). We used the mean square error (MSE) between each of the model’s predicted learning curves and the participants’ actual learning curves to measure model performance. A one-way ANOVA showed that there was a significant difference in MSE across these three models (*F*(2) = 7.47, *p* = 0.002, *η*^2^ = 28.22). Post hoc (2-tailed) paired t-tests indicated that the model using individual Gamma and individual motor noise (MSE = 11.29 ± 13.09) explained significantly more variance than the alternative Model 1 (MSE = 20.41 ± 21.18, t(19) = 3.56, p = 0.002, d = 0.80), and the alternative Model 2 (MSE = 26.88 ± 32.32, t(19) = 2.96, p = 0.008, d = 0.66). However, these two alternative models were not significantly different from one another (t(19) = 1.67, p = 0.11, d = 0.37). This indicates that both the Gamma (Γ) and motor noise parameters were important for the model to best predict participant behaviour in the MO task.

### Behavioural analysis: Decision-making task with ‘motor noise’

**Fig 10:**
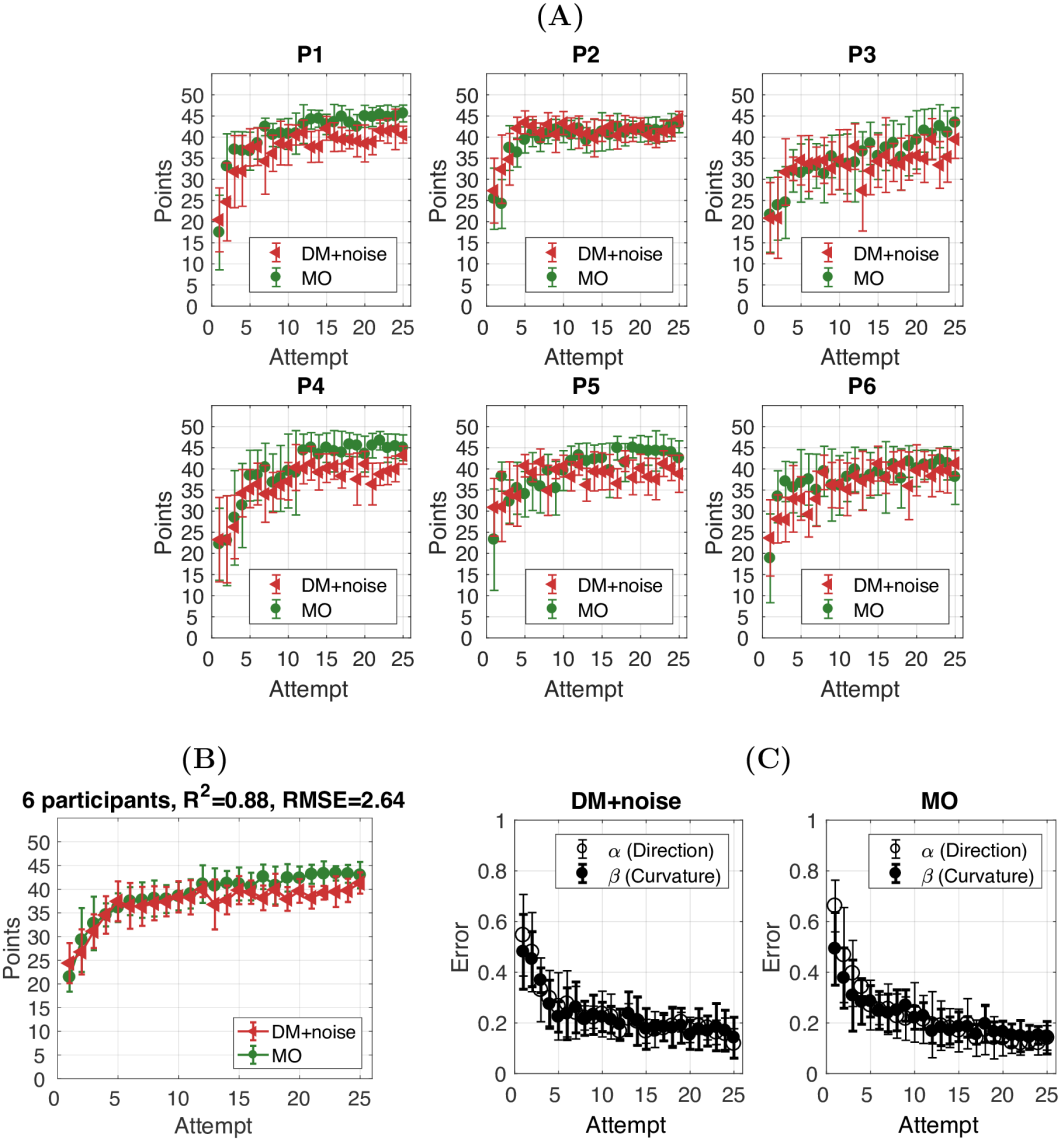
Experiment 2: Decision-making with motor noise. **(A)** Learning curves for the DM+noise (red) and MO (green) tasks for each participant. The *R*^2^ between the DM+noise and MO task is provided. **(B)** Average learning curves across 6 participants. **(C)** Error in *α* and *β* (y-axis) plotted against the number of attempts (x-axis) in the DM+noise and the MO task. Error bars in all panels represent 95%CI across participants.

The previous modelling showed that individual performance in the decision-making task (parameter Γ) and motor noise task were both critical for predicting individual performance in the reaching task. Next, we examined whether participant performance in the decision-making task would become similar to their performance in the reaching task if their individual ‘motor noise’ was added to the feedback they received during decision-making. We recruited a further 6 participants for Experiment 2. In this experiment, we asked each participant to complete the same reaching and motor noise task as in the previous experiment. However, for the decision-making task, the feedback score provided after each attempt (i.e., clicking on a cell) now included noise parameters that were equivalent to the level of noise/uncertainty observed in the motor noise task for each participant (DM+noise). For example, when a cell [*α*_1_, *β*_1_] is selected and the target is [*α*_*T*_, *β*_*T*_], the feedback score is determined by two errors: |*α*_1_ − *α*_*T*_| + *noise*_*α*_ and |*β*_1_ − *β*_*T*_| + *noise*_*β*_, instead of |*α*_1_ − *α*_*T*_| and *β*_1_ − *β*_*T*_| as in the previous experiment. Two motor noise parameters: *noise*_*α*_ and *noise*_*β*_ were measured in the motor noise task. Fig 10 shows that participant learning in the decision-making task with ‘motor noise’ (DM+noise) and the reaching task (MO) was now identical (*R*^2^ = 0.88, rmse = 2.64, Fig 10B). Once again, three two-way repeated measures ANOVAs were conducted on fitted exponential parameters a, b and c. Unlike Experiment 1, we found that the error reduction was not significantly different either across *α* and *β* or across tasks (Fig 10C; S2 Table)

### Gains and losses

As we said in the introduction, we were also interested in whether trial-by-trial motor performance could be predicted as a function of gains and losses. It has been suggested by a number of authors that the effects of gains and losses maybe be elucidated via trial-by-trial analysis of choice behaviour, as the outcomes of previous choices have been shown to affect subsequent decisions [33, 34]. For example, in a sequential tree search task, it has been shown that participants are more likely to curtail any further evaluation of a branch as soon as they encountered a large loss [35]. Another example of these local influences on choice behaviour is a tendency to repeat the same behaviour following a gain, coupled with a bias to switching behaviour after a loss [36].

**Fig 11:**
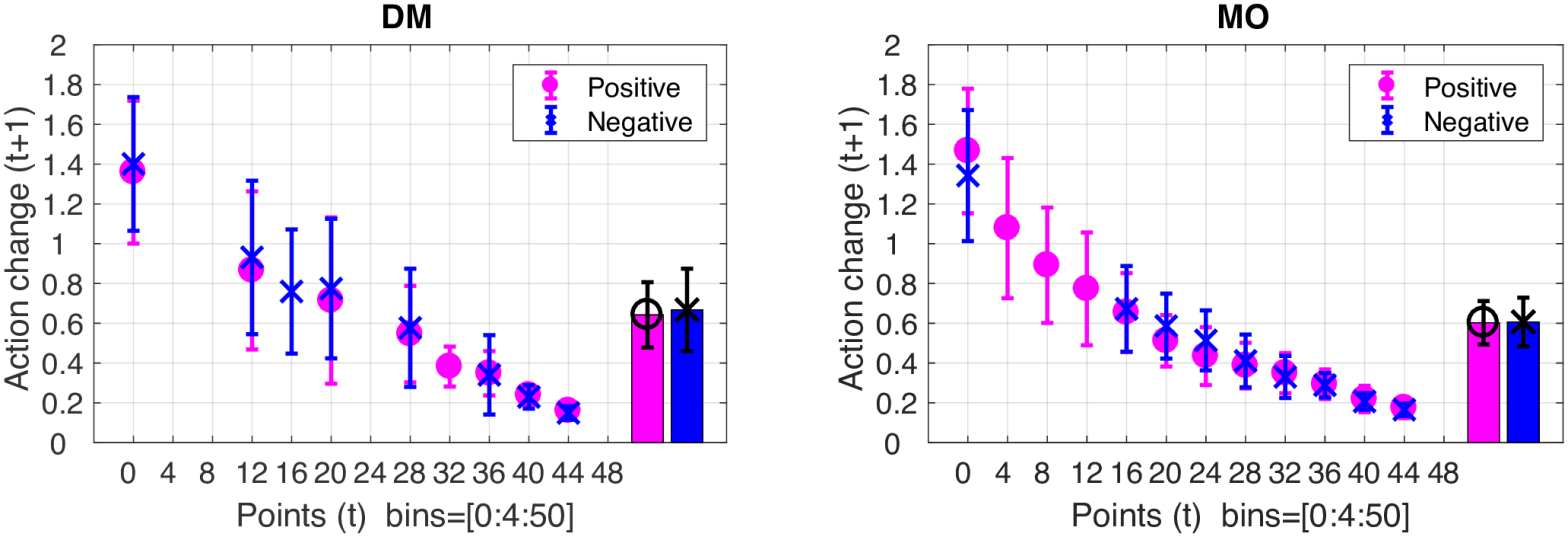
Action change following positive and negative feedback. Action change on attempt (*t* + 1) following a score on attempt *t* in the positive (pink circle) and negative (blue cross) feedback conditions for the DM (left) and MO task (right). Mean action change for each participant for the positive condition (pink bar with circle) and the negative condition (blue bar with cross) in the DM task (left) and the MO task (right).

In Experiment 1, gains and losses are operationalised as positive and negative feedback. Here, we examine the degree of action change on attempt *t* + 1 after receiving a certain score on attempt *t*. As mentioned (page 6), the action change was defined as the Euclidean distance between two actions. First, we compared action changes between positive and negative feedback conditions. The action change following a score of 10 (10 points above the minimum point 0) in the positive condition was compared to the action change following -40 (10 points above the minimum point -50) in the negative condition. Paired t-tests revealed no significant difference between positive and negative conditions for either the DM task (t(23) = -1.00, p = 0.32, d = -0.21; Bars in Fig 11) or the MO task (t(23) = -0.26, p = 0.79, d = -0.05; Bars in Fig 11). Model predictions were given in Fig 9.

Next, we considered whether gains and losses are better measured relative to a reference point. Prospect theory suggests that gains and losses are measured relative to a reference point that may shift with recent experience [18, 38]. It follows that within the current study, participants may have thought of gains and losses relative to the best score achieved so far while searching for the current target. For example, a participant who received a score of 22 on their 8th attempt might see this as a loss of 13 given that their best score so far (on attempt 4) was 35. Therefore, during the 25 attempts, the current best score could be thought of as the current reference point. A score that was better than the reference point can be defined as a gain, and a score worse than this reference point a loss.

**Fig 12:**
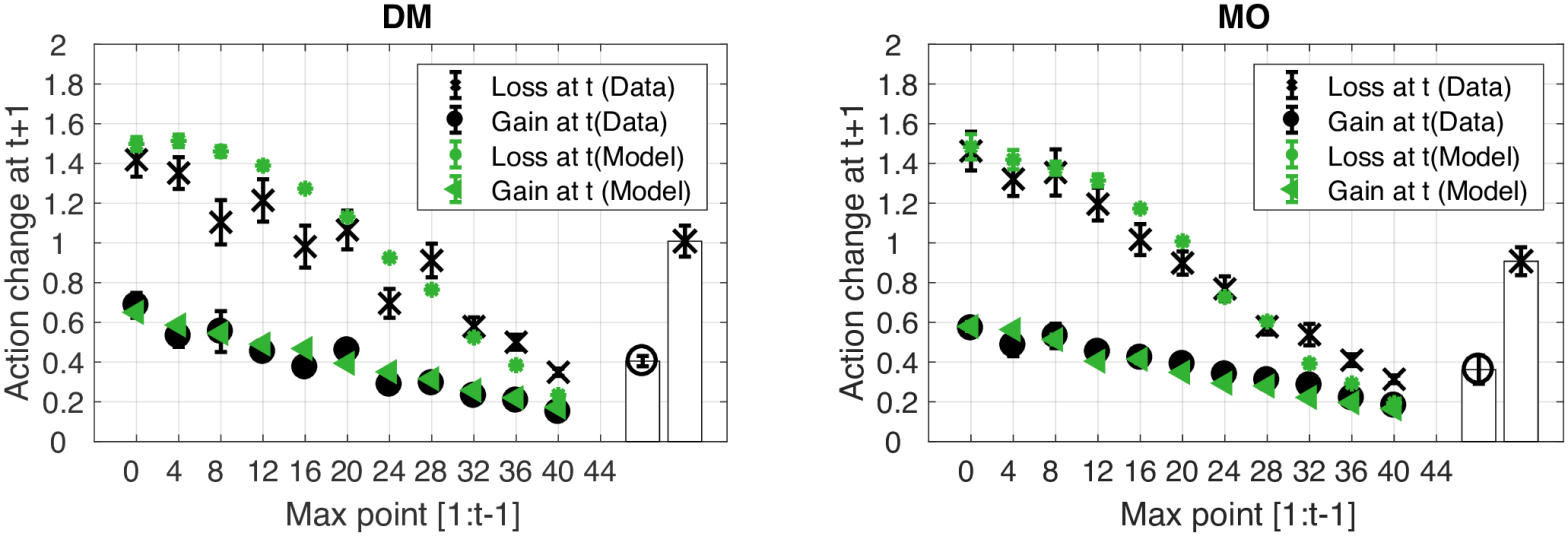
Action change relative to the current highest score (the reference point). Action change on attempt *t* + 1 was plotted as a function of the maximum points achieved up to *t* − 1 (the reference point) for the DM task (left panel) and MO task (right panel). If the score on attempt *t* was greater than the reference point, then the action change at *t* + 1 was considered as an action after a gain (black circle). If the score on attempt *t* was smaller than the reference point, then the action change after this score (*t* + 1) was considered as an action after a loss (black cross). Model (green) predictions are also provided. The bars represent the mean action change in the DM task (left panel) and the MO task (right panel). Error bars represent 95% CI across participants.

Participant data was pooled across the positive and negative feedback conditions by transforming a negative score into its positive equivalent. We investigated action change on attempt *t* + 1 as a function of the maximum points achieved up to *t* − 1 (the reference point). A gain was a score that was better than the reference point on attempt *t*, and a loss was a score that was worse or equal to the reference point on attempt *t* (Fig 12). Paired t-tests indicated that the action change following a loss was statistically greater than the action change following a gain in both the DM task (*t*(23) = 11.39, *p* < 0.001, *d* = 2.32; Bars in Fig 12 Left) and MO task (*t*(23) = 12.18, *p* < 0.001, *d* = 2.49; Bars in Fig 12 Right). The model predicted this behaviour in both the DM task (*R*^2^ = 0.91, RMSE = 0.13; Fig 12) and MO task (*R*^2^ = 0.96, RMSE = 0.10; Fig 12).

This suggests that participant sensitivity to gains and losses was possibly independent of the positive and negative feedback conditions but in fact related to a shifting reference frame determined by their current best score.

## Discussion

### Summary

Our goal was to examine whether explorative motor learning [13, 14] and decision-making could be modelled as the (approximately) optimal solution to a Partially Observable Markov Decision Process [25] bounded by noisy neural information processing. To achieve this, we studied performance during an explorative motor learning task [13] and a decision-making task which had a similar underlying structure with the exception that it was not subject to motor noise. The solution to the defined POMDP explained 0.94 of the variance in the decision-making task, and 0.76 of the variance in the explorative motor learning task. Importantly, we did not fit the model to the motor learning data but predicted it based on parameters derived from the decision-making task and a separate motor noise task. In addition, the model was also able to explain (1) varying performance across different target trajectories, (2) the magnitude of action change after different scores, and (3) the differences in the magnitude of action change between gains and losses.

### Explorative motor learning and decision-making

A key contribution of the work reported here is to furthering our understanding of the relationship between motor learning and decision making. In the reported studies, the decision-making task allowed measurement of a participant’s ability to make use of information in previous attempts. Participants with a high likelihood uncertainty were less able to integrate this information and were slower learners. Participants with a low likelihood uncertainty were more able to integrate information and were faster learners. Given an equivalent level of motor noise, participants who were faster learners in the decision-making task were also faster learners in the reaching task.

We can draw this conclusion because of the modelling approach that we used. We used the likelihood uncertainty parameter estimated from the decision-making task, and the individual motor noise estimated from the motor noise task, to predict motor learning behaviour. Importantly, we found that the model performed significantly worse when averaged parameters (across all participants) were used rather than parameters derived from each individual’s behaviour. This suggests that taking into account individual performance during both the decision-making and motor noise tasks was important for explaining behaviour during the explorative motor learning task. Finally, we showed that performance during the decision-making task was similar to performance in the reaching task if motor noise was added to the decision-making task’s feedback. This provides strong empirical evidence for the predicted relationship between explorative motor learning, decision-making and motor noise within our model.

Although the decision-making task was designed to have a similar underlying structure to the reaching task there were still differences. For example, unlike the explicit visual cues of orthogonally organised actions in the decision-making task, the relationship between the two parameters was less intuitive in the reaching task. It is possible that this could have led to these parameters being treated more dependently in the reaching task. For instance, the errors of these two parameters were correlated during the ‘motor noise’ task (*r* = 0.52, *p* = 0.008). However, Dam et al., (2013) [13], who used a near identical motor learning task, showed that participants were able to isolate direction and curvature so that they only altered the parameter being currently rewarded. We believe our results suggest that participants treated the parameters in a similar fashion within the reaching task and decision-making task. For example, the rate of error reduction for the two dimensions was similar within each task, indicating that participants explored both parameters simultaneously, while also implying a comparable strategy across both tasks. Another potential difference was that there were clearly defined discrete action options (grid-design) in the decision-making task. It has previously been shown that there are limits to the sensory and motor system’s ability to distinguish endless continuous options [42, 43]. For example, our ability to distinguish two shades of grey is limited rather than continuous. This suggests that the motor learning task may also have involved a set of discrete action options. However, our tasks were not designed to measure participant ability to distinguish between trajectories that varied in direction and curvature. Therefore, it was not possible to define what these discrete action options could have been during the motor learning task. Future work could examine whether the ability of the decision-making task to predict explorative motor learning is improved by creating a grid-size which directly reflected participant’s ability to distinguish trajectories with different curvature and directions.

It was also clear that our model did not fully explain motor learning behaviour. For example, the model predicted faster learning rates and higher plateaus than what was achieved by the participants for a number of trajectories in the reaching task. These trajectories had large amounts of curvature and also began on one side of the central line and finished on the opposite side. These elements appeared to make the trajectories more difficult for the participants than the model. One might argue that these types of trajectories are less likely to be performed in everyday life and therefore are more difficult to find through exploration [44, 45]. Alternatively, such ‘two-direction’ movements may be more difficult to execute. To improve the model’s performance, future work could utilise a more sensitive measure of motor noise by obtaining a curvature and direction noise measurement for each of the trajectories examined.

### Exploration and motor noise for motor learning

Variability in movement is a fundamental component in motor behaviour. It is caused by numerous factors including planning, sensory and neuromuscular noise [46]. Researchers often categorise variability into two sources: exploration and motor noise. Exploration represents the variability which results from ‘intentional’ exploration of different actions [15]. While motor noise represents the variability observed when attempting to repeat a single action [24]. Previous work has examined the differential role of exploration and motor noise in motor learning [14, 15, 46]. For example, Wu et al., (2014) found a positive relationship between motor noise and motor learning [14]. This is in contrast to our, and others [15], results which showed a strong negative relationship between motor noise and the rate of motor learning. However, this difference in findings might be explained by differences in experimental design. Whereas Wu et al.’s (2014) participants were provided with visual guidance for a trajectory (which differed from the reward trajectory), our participants were provided with a reward signal but not provided with visual guidance. In Wu et al.’s (2014) task success was based on the similarity between a participant’s attempted path and a reward shape that was similar but independent from the guided shape. Therefore, participants would have initially attempted to execute the guided shape; individuals with higher motor noise would deviate from this shape to a greater degree and thus be more likely to find the underlying rewarded shape. In contrast, in our study learning was achieved by determining the relationship between attempted actions and their associated points where the points were based on the executed action not the intended action. As motor noise represented the gap between the intended and executed action, the greater the motor noise, the more difficult and slower the process of learning the relationship between actions and points was likely to be. He et al., (2016) have shown that motor variability can have a complicated relationship with learning: positive, negative and neutral [46]. They emphasised that it is important to consider the relationship between motor noise and learning in a task-specific manner [46].

### Neural basis of explorative learning

Defining explorative motor learning as a sequential decision-making task suggests that this form of motor learning could be dependent on brain areas more commonly associated with cognitive decision-making such as the frontal cortex and basal ganglia [47–54]. However, it is unclear how varying levels of motor noise alter this type of ‘cognitive’ learning. Although with a very different task, it has recently been shown that explorative motor learning is impaired in patients with cerebellar damage who show increased levels of motor noise [15]. One suggestion is that the cerebellum predicts the sensory state of an action and feeds it to the basal ganglia [55] or frontal cortex [56], which in turn estimates the value of the new state through reinforcement processes. Without the cerebellum, predicted action outcomes may be poorly represented, or even unknown, and so linking them to reward values would be more difficult. This increased (motor) noise in predicting movement outcomes could lead to greater uncertainty with respect to reward based predictions and thus a reduced ability, or reluctance, to adapt behaviour [57, 58]. Although we are not suggesting that participants in our study, who displayed increased motor noise had a damaged cerebellum, such a neural mechanism could readily explain our results.

### Gains and losses

Although a great deal of research has investigated cognitive decision-making [18, 59, 60], only recently have researchers begun to examine motor-based decision-making [17, 19]. Some studies have shown humans perform optimally when making motor decisions, in contrast with markedly sub-optimal and biased performance in economic decision-making [20, 61]. In contrast, other studies have revealed similar sub-optimal behaviour across motor and cognitive decision-making tasks [20, 62]. One of the most influential findings in decision-making is that people behave asymmetrically with gains and losses (e.g., loss aversion, [18]). When initially comparing performance with positive or negative feedback, we observed no observable differences. However, one of the difficulties in defining gains and losses during a dynamic learning process is that the definition of gains and losses is highly dependent on previous experience [18, 37, 63]. This is known as a reference point in economic decision-making tasks, with the value and importance of the reference point being altered by task instructions and feedback [18, 63]. For example, providing a running total on the screen causes participants to make choices based on this reference point rather than making an independent decision based on the current trial [37]. Therefore, we decided to collapse the data across positive and negative feedback and instead look at participant behaviour in terms of whether a trial was better (gain) or worse (loss) than the maximum (best) achieved so far. This reference point was chosen as it reflected the instructions of the task. By defining the score relative to this dynamic reference point, we found participants made substantially larger changes in behaviour following a loss compared to a gain. This suggests that when comparing positive (reward) and negative (punishment) feedback in motor learning [23, 64, 65] a clear understanding of the reference point being used by the participants is crucial. In other words, it should not be assumed that positive feedback is always a gain and negative feedback is always a loss [37]. Interestingly, our model shows that the asymmetric response of participants to gains and losses is not irrational. Rather, participants are responding optimally given their likelihood and motor uncertainty. Essentially, what the model does is discover the bounded optimality of the loss-shift/win-stay heuristic.

### Partially Observable Markov Decision Process

The reported model builds on preceding work that has explored the use of POMDP, and related models, for explaining various aspects of human decision-making [27, 28, 31, 32, 39–41]. Framing the model as a POMDP allowed us to calculate the (approximately) optimal strategy given certain pre-defined constraints. It is this calculation that allows the model to make predictions of behaviour in the reaching task given parameters set to the decision-making task and the motor noise task. In other words, the strategy is determined by the optimisation and not by the theorist picking a strategy that fits the data. The behaviour predicted by the optimal policy is therefore the rational behaviour given the constraints. The POMDP framing thereby serves the goal of drawing a causal relationship between the theoretical constraints and the behaviour (assuming rationality, [32]). If theorists are to progress in explaining human behaviour then they must move away from fitting models to the data that they are trying to explain [31]. The POMDP framing supports such a move.

One advantage of a POMDP framing is that models framed in this way are readily falsifiable. The modelling involves specifying the states, actions, observations and rewards (a POMDP problem), computing the optimal policy, and comparing the predicted behaviour to the observed human behaviour. If there are discrepancies between the predictions and the observed human behaviour, alternate theories of the constraints can be explored, the model can be refined, and the process repeated. This iterative process leads to the assertion of a set of theoretical constraints that would lead a rational human generating the observed behaviours [29, 32, 66].

However, in practice, POMDPs are often computationally intractable to solve exactly. Computer scientists have developed methods that approximate solutions for POMDPs. More recent work has made use of sampling techniques, generalisation techniques and the exploitation of the problem’s structure to extend POMDP into large domains with millions of states [67]. Importantly, this work provides a basis to attempt more comprehensive techniques in order to better approximate the optimal solution for this task.

### Conclusion

In conclusion, we modelled behaviour during an explorative motor learning task and a decision-making task with similar underlying structure using a Partially Observable Markov Decision Process (POMDP). The model was able to predict performance in motor learning by using parameters estimated from the decision-making task and a separate motor noise task. This suggests that explorative motor learning could be considered as a sequential decision-making process that is adjusted for motor noise. This work reinforces the view that the mechanisms which control decision-making and motor behaviour are highly integrated and raises interesting questions regarding the neural origin of explorative motor learning.

## Methods and models

### Ethics statement and subjects

The study was approved by Ethical Review Committee of the University of Birmingham, UK, and was in accordance with the declaration of Helsinki. Written informed consent was obtained from all participants. Participants were recruited through online advertising and received monetary compensation upon completion of the study. Thirty-two healthy individuals were recruited for the two experiments. All were naïve to the task, had normal/corrected vision, and reported to have no history of any neurological condition (Mean age: 26.46 ± 5.96; 17 females; 25 right handed). Twenty-six participants participated in Experiment 1 (however, one withdrew during the experiment due to personal reasons; another one did not finish the experiment due to equipment malfunction). Six new individuals participated in Experiment 2.

### Experiment 1

#### Procedure

All participants completed both the motor learning (reaching) task and the decision-making task; the order was counterbalanced across the participants. In addition, all participants completed the motor noise measurement task prior to the main motor learning task.

#### Explorative motor learning task

Participants were seated with their heads supported by a chin-rest (Fig 1A), looking down at a horizontal (65cm × 40cm) mirror, which reflected task-related stimuli from a computer screen. The mirror blocked direct observation of the index finger, which was instead represented on the mirror via a circular green cursor (0.25cm diameter). Index finger position was recorded at a sampling rate of 120Hz by a Fastrak motion tracking system (Polhemus, USA) through a custom Matlab (Mathworks, USA) program. Participants were asked to draw trajectories (Fig 1B) by sliding their index finger from a central start position (1cm^2^ square) across the surface of a desk towards a target line positioned 15cm (Y-direction) in front of the start position (the thick black line in Fig 1B). This target line had a length of 50cm (X-direction), with the end of movement being defined as the point at which the index finger (represented by the green cursor) hit this line. Each participant attempted to match 24 different, invisible target trajectories that varied in both direction and curvature [13].

All participants experienced the same set of 24 target trajectories that were given in a random order (Fig 1C). The shapes of the trajectories were defined by:

x=αy+βsin(πy) (1)

where the y-coordinates (Y) represented the reaching depth, which was 15 cm, and the x-coordinates (X) of the trajectory were determined by two parameters, direction (*α*) and curvature (*β*). The 24 target trajectories were formed by a Cartesian product of *A* = [-0.8,-0.4,0.4,0.8] and *B* = [-0.9,-0.6,-0.3,0.3,0.6,0.9]. The Cartesian product includes a set of 24 ordered pairs (*α*, *β*), where *α* ∈ *A*, *β* ∈ *B*, each of which is a target parameter pair. All target trajectories were confined within a quadrangular table-top space of 46.5cm in width and 15cm in depth (Fig 1C). Participants were permitted to generate trajectories within a space of 50cm in width and 15cm in depth, with this being defined by an outer white square displayed on the mirror (Fig 1A and 1B). Importantly, these 24 targets were generated to be evenly distributed across the workspace, so that learning was minimally affected by a target location bias (Fig 1C).

Participants made 25 attempts to approximate each desired target trajectory (Fig 1B). Each attempted trajectory resulted in a score that indicated the proximity of the attempted trajectory to the target trajectory. The 24 target trajectories were randomly divided into two feedback conditions (12 of each): a positive feedback condition and a negative feedback condition. In the positive feedback condition, points ranged from 0 to 50, with greater magnitude indicating greater similarity between the attempted and target trajectory. In the negative feedback condition, points ranged from −50 to 0, with greater magnitude indicating reduced similarity between the attempted and target trajectory. Hence, the goal for the positive feedback condition was to achieve 50 points, whereas for the negative feedback condition it was to achieve 0 points (i.e., avoiding losing points). These points were directly related to monetary incentive (2 points were equivalent to 1 pence). Participants were told which of the two feedback conditions they were in at any time. Participants were informed that they would receive the highest reward or lowest punishment that they achieved from the trajectory attempts for each target trajectory. Each target search was terminated by either finding the hidden trajectory, and so obtaining a maximal score, or by reaching attempts. Movement duration was defined as the time between the cursor leaving the start position and it hitting the target line (Fig 1B). If the movement duration was < 700ms or > 1500ms, the attempt was deemed invalid, leading to the score being withheld and the trial being repeated. Hence, a total of 25 valid attempts were permitted per target trajectory. After each valid attempt, participants were presented with the feedback. Positive feedback was presented in yellow text as ‘You won xx points.’, while negative feedback was presented in red text as ‘You lost xx points’. For invalid trials, no score feedback was provided and instead the home square turned from white to either red (if the movement was too slow) or green (if the movement was too fast).

To determine the score, each attempted trajectory was fitted to Eq 1 in order to obtain an estimate of its direction and curvature parameters. The error in the direction was: Δ*α* = |*α*_*target*_ − *α*_*attempt*_|, and the error in curvature was: Δ*β* = |*β*_*target*_ − *β*_*attempt*_|. The feedback score (Eq 2) was determined by the total error (*ε*) of the attempt along both dimensions: *ε* = 1 − (0.5 × Δ*α* + 0.5 × Δ*β*) [13]. Therefore, the score (points) in the positive feedback condition were integers in [0, 50]. The score (points) in the negative feedback condition were integers in [-50,0].

$$S=\left\{\begin{array}{cc} max(0,||50\times \varepsilon ||), & \text{if}\;\text{condition = positive}\\ -(50-max(0,||50\times \varepsilon ||)), & \text{if}\;\text{condition = negative} \end{array}\right.$$ (2)

#### Motor noise measurement task

Unlike the main learning task where the target trajectories were hidden, a series of trajectories was displayed on the screen. All participants experienced the same set of 10 trajectories which were given in a random order. As in the main motor learning task, each trajectory was defined by a parameter pair (*α*, *β*) as in Eq 1. These trajectories were formed by a Cartesian product of *A* = [-0.7,0.7] and *B* = [-0.8,-0.4,0,0.4,0.8], which contains a set of 10 ordered parameter pairs (*α*, *β*), where *α* ∈ *A*, *β* ∈ *B*. These trajectories were not repeated during the main experiment. During this task, the participants could see a circular green cursor that tracked the index finger.

For each displayed trajectory, the participants were asked to trace it within a time window that was identical to the one used in the main experiment (> 700ms and < 1500ms). Only the movements that were within this time window were deemed valid traces. No score feedback was provided during the motor noise task. For each invalid trace, the home square turned from white to either red (if the movement was too slow) or green (if the movement was too fast). Five valid traces were performed for each trajectory (Fig 4A).

To measure the execution error due to motor noise, each valid trace was fitted to Eq 1 to obtain a pair of estimated direction and curvature parameters. By comparing the estimated direction and curvature parameters with the target parameters, we obtained one direction error and one curvature error for each valid trace. Therefore, we had 5 pairs of errors for each target (5 valid traces). Each participant was asked to trace 10 target trajectories. Hence, we collected 50 errors in the direction and 50 errors in the curvature. For each participant, we calculated the standard deviation across the errors in the direction and curvature parameters and used these two standard deviations as our measure of their motor noise in the direction and curvature dimensions, respectively.

#### Decision-making task

Participants in this task were interacting with an interface using a computer mouse. The interface was designed using Matlab and displayed on a desktop PC. The interface consisted of a two-dimensional grid, in which there were 21 × 21 cells (Fig 1D). The horizontal and vertical dimension were defined with two parameters: *α* and *β* respectively, akin to the direction and curvature parameters in the reaching task. Both parameters ranged from -1 to 1 with 0.1 increments. The parameter values were assigned to the cells in a spatially ordered manner. Specifically, the cells in the same row had the same *β* values, but with *α* values ordered from -1 to 1 with an 0.1 increment (from left to right, Fig 1D); the cells in the same column had the same *α* values, but with *β* values ordered from -1 to 1 with an 0.1 increment (from bottom to top, Fig 1D). Therefore, each cell of the grid corresponded to a unique combination of the two parameters. When one of the cells (i.e., one parameter pair) was chosen as a target cell, the score associated with each of the cells was then calculated based on Eq 2. That is, the score for decision-making task was calculated using the same function as in the reaching task. Once a cell was chosen (mouse-clicked), an associated score would appear in the feedback window at the top of the screen.

Participants were asked to search for a series of 24 hidden target cells by exploring the cells on the grid. The participants were told that the rows and columns represented different values in an ordinal space. Similar to the reaching task, the 24 target cells were randomly divided into two feedback conditions (12 of each): a positive feedback condition and a negative feedback condition. Again participants were informed that they would receive the highest reward or lowest punishment that they achieved from the 25 attempts for each target cell. Each target search was terminated by either finding the hidden target cell, and so obtaining a maximal score, or by reaching 25 attempts. The 24 target cells were formed by the ordered pairs [*α*, *β*], where *α* ∈ *A*, *A* = [-1,-0.5,0.5,1], *β* ∈ *B*, *B* = [-1,-0.6,-0.2,0.2,0.6,1]. These pairs were intentionally different to the reaching task however covered the same workspace where both the two parameters ranged from -1 to 1 with 0.1 increments.

Therefore, a target cell in the decision-making task could be considered identical to a target trajectory in the reaching task as they were both defined by a combination of *α* and *β* within a similar workspace. In addition, the score for the decision-making task was calculated using the same function as in the reaching task (Eq 2). Therefore, we believe this provided two explorative learning tasks that were analogous except that the decision-making task did not involve motor uncertainty/noise between the planned and executed behaviours.

#### Payoff scheme

At the onset of the experiment, participants were given £5 in cash. They were informed that their final payoff would be increased by the positive points obtained across both tasks, and decreased by the negative points across both tasks. Although participants were informed of their best score at the end of each target trajectory/cell, they were not given a running total until the end of the experiment. The experiment lasted around 90 minutes. Their average points achieved for the decision-making task was 595 ± 8.71, and 469 ± 81.0 in the reaching task. The average total payment was £10.32 ± £0.42.

### Experiment 2

All participants first completed the motor noise measurement task. This task was the same as in Experiment 1. Participants then completed the motor learning (reaching) task, followed by a different version of the decision-making task (i.e., with motor noise added into the feedback score). The reaching task was the same as in Experiment 1 except that the participants were asked to find half of the target trajectories (i.e. 12) with positive feedback (points ranged from 0 to 50). That is, only the positive condition in Experiment 1 was replicated in Experiment 2. These 12 target trajectories were formed by [*α*, *β*] pairs shown in Table 2.

**Table 2:** Target parameters used in the MO and the DM+noise task in Experiment 2.

Targets	MO	DM+noise
[*α*, *β*]	[-0.8,-0.6], [-0.8,-0.3], [-0.8, 0.3] [-0.4,-0.6], [-0.4,-0.3], [-0.4, 0.3] [0.4,-0.3], [0.4, 0.3], [0.4, 0.6] [0.8,-0.3], [0.8, 0.3], [0.8, 0.6]	[-0.8,-0.6], [-0.8,-0.3], [-0.8, 0.3] [-0.4,-0.6], [-0.4,-0.3], [-0.4, 0.3] [0.4,-0.6], [0.4,-0.3], [0.4, 0.3] [0.8,-0.6], [0.8,-0.3], [0.8, 0.3],

In the decision-making task, participants were asked to find 12 hidden target cells (Table 2) with positive feedback (points ranged from 0 to 50). Unlike the decision-making task in Experiment 1, the ‘motor noise’ was added to the feedback score as follows. Recall that, for each participant, we calculated the standard deviations of the direction errors and the curvature errors during the noise measurement task. We used these two standard deviations, *σ*_*dir*_ and *σ*_*cur*_, as the measure of their direction motor noise and curvature motor noise respectively. For a clicked cell with parameters [*α*_*attempt*_, *β*_*attempt*_], the feedback score was based on $\left[\alpha_{attempt}^{\prime },\beta_{attempt}^{\prime }\right]$, where $\alpha_{attempt}^{\prime }=\alpha_{attempt}+N\left(0,\sigma_{dir}\right)$, and $\beta_{attempt}^{\prime }=\beta_{attempt}+N\left(0,\sigma_{cur}\right)$. That is, the feedback score for the cell: [*α*_*attempt*_, *β*_*attempt*_] was calculated using the same score function as in Experiment 1 (Eq 2) given the cell $\left[\alpha_{attempt}^{\prime },\beta_{attempt}^{\prime }\right]$. The instructions given in this experiment were the same as in Experiment 1.

### Model for the decision-making task

The decision-making task is formulated as a POMDP as follows [25]. There is a set of states S, each of which corresponds to an event in which the target is one of the cells in the grid (Fig 1D). At any time step *t*, the environment is in a state $s_{t}\left(i,j\right)\in S$, where *i* and *j* indicate the target location in the grid, *i* ∈ [1: 21], *j* ∈ [1: 21]. Therefore, there are 441 (21 × 21) states in the state space S. As the experiment to be modelled, the task is divided into episodes; one of the cells is randomly chosen as the hidden target on each episode; each episode consists of 25 time steps (attempts) to find the hidden target cell. That is, the environment is in one of the states; and the state is not directly observable. On each time step within one episode, the model chooses an action. Each action represents an event of clicking one cell in the grid, a(i,j)∈A, *i* ∈ [1: 21], *j* ∈ [1: 21]. Therefore, there are 441 (21 × 21) actions available on each time step. After taking an action *a*, the environment transitions from state *s* to a new state *s*′ according to the transition function $T\left(s,a,s^{\prime }\right)=Pr\left(s^{\prime }|s,a\right)$. Note that the underlying state of the environment (i.e. the target cell) remains unchanged within each episode. Therefore, the transition function T equals to 1 only when *s*′ = *s*; it equals 0 otherwise. That is, the state transition matrix is the identity matrix. After taking the action *a*, the model also receives two signals from the environment: an observation o∈O and a reward r∈R (cost if the value is negative). In our task, the observation and reward are equal, which is the feedback score (points between 0 and 50 (or [-50:0]). The feedback score is calculated based on the hidden target location (i.e. state) and the clicked cell (i.e. action) as in the experiment (Eq 2).

For the decision-making task, one assumption is that participant performance was constrained by the fact that they were naïve to the underlying equation used to generate the score. We model this with a likelihood uncertainty parameter Γ (‘Gamma’), which represents the uncertainty the score would receive for the current action if a target cell is in a certain location. Specifically, the observation function is the conditional density of the observation given the true state of the environment and the action, *p*(*o*_*t*_|*s*, *a*_*t*_). This function is normally distributed around the true score (based on Eq 2); the standard deviation of this normal distribution is the likelihood uncertainty parameter Γ:

$$p\left(o_{t}|s,a_{t}\right)∼N\left(trueScore\left(s,a_{t}\right),\Gamma \right)$$ (3)

Given the defined POMDP, an algorithm is then used to acquire the optimal control policy (an approximate solution was used in our model). This is the control policy that maximises the expected sum of rewards over 25 steps:

$$E[\sum_{t=1}^{25}r\left(t\right)]$$ (4)

where *r*(*t*) is the reward on time step *t*, *E* represents the expected value over all the uncertainty in the task performance. Hence, for example, if the model is certain about the state (i.e., target cell), then the control policy (for action selection) becomes trivial (i.e., clicking the target cell on each of the 25 steps), thus the expected reward in this situation would be: $E[\sum_{t=1}^{25}\left(50\right)]=50$, given that the reward (points) obtained for clicking on the target cell is 50. As the model is unsure of the target location (i.e., the model does not directly observe the underlying state of the environment), it must rely on its history of actions and observations. This history is used to estimate the current (unobserved) state (i.e. to estimate where the target is given the action/observation history). This history information is succinctly captured by the *belief state*. The belief state is a posterior probability distribution over the state space given past observations and actions. The action selection is thus then based on the belief state. Our approach involves a Bayesian belief update for state estimate and a control part for action selection (Fig 6). The control part is to select actions, so as to maximise the expected reward (approximately).

#### Bayesian belief update

Specifically, the states are discrete in our task, S=s(i,j),i∈[1:21],j∈[1:21]. A belief state is therefore represented as a matrix of probabilities whose size is the same as the state space. The belief state at time step *t* is: $b_{t}\left(i,j\right)\in B$, *i* ∈ [1: 21], *j* ∈ [1: 21]. Each element *b*_*t*_(*i*, *j*) represents the posterior probability of the state *s*(*i*, *j*) after the history of actions, *a*_1,2,3,…,*t*−1_, and observations *o*_1,2,3,…,*t*−1_. The initial belief state *b*_0_ (red arrow in the right of Fig 6) was assumed to be an uniform distribution across the state space. That is, without any evidence, the model believes that the environment is equally possible to be in one of the states.

The update process is as follows. At *t*, an action, *a*_*t*_, is taken, which causes the environment to transition from state *s* to state *s*′ with probability *T*(*s*′ ∣ *s*, *a*) (the transition function). After reaching *s*′, one observation, *o*_*t*_, is received with probability *p*(*o*_*t*_ ∣ *s*′,*a*_*t*_) (the observation function). The belief state, *b*_*t*_, is obtained given the action *a*_*t*_, the observation *o*_*t*_, and the previous belief *b*_*t*−1_, as in Eq (5) below.

$$b_{t}\left(s^{\prime }\right)=\frac{\sum_{s\in S}b_{t-1}\left(s\right)\times T\left(s^{\prime }|s,a_{t}\right)\times p\left(o_{t}|s^{\prime },a_{t}\right)}{\sum_{s^{\prime }\in S}p\left(o_{t}|s^{\prime },a_{t}\right)\sum_{s\in S}T\left(s^{\prime }|s,a_{t}\right)b_{t-1}\left(s\right)}$$ (5)

As mentioned above *T*(*s*′|*s*, *a*_1_) = 1 only if *s*′ = *s*, and 0 otherwise, Eq (5) can be simplified as Eq (6):

$$\begin{array}{cc} b_{t}\left(s\right) & =\frac{b_{t-1}\left(s\right)\times p\left(o_{t}|s,a_{t}\right)}{\sum_{s\in S}p\left(o_{t}|s,a_{t}\right)b_{t-1}\left(s\right)}\\ & \propto b_{t-1}\left(s\right)\times p\left(o_{t}|s,a_{t}\right) \end{array}$$ (6)

where the likelihood *p*(*o*_*t*_|*s*, *a*_*t*_) denotes the conditional density of the observation given the true state of the environment and the action. As mentioned, *p*(*o*_*t*_|*s*, *a*_*t*_) is normally distributed around the true score with the standard deviation Γ (Eq 3) (The implementation details can be found in S1 Text and also in online code).

#### Action selection

The belief state is the best estimate of the current state given the observation/action history, and the action is chosen by the optimal control policy (Fig 6). That is, the POMDP is now a belief-state MDP. Any solution that solves MDP could be, theoretically, used to solve this problem, including Q-learning [68], Value/Policy Iteration [16]. However, in practice, POMDPs are often computationally intractable to solve exactly, so computer scientists have developed methods that approximate solutions for POMDPs. We used one of the approximated solutions called QMDP [69, 70]. The logic is that the value of each action given a belief, Q(b,a),b∈B,a∈A, is equal to the sum of the expected reward after taking this action in each state, Q(s,a), multiplied by the probability of the agent being in that state, b(s):

$$Q\left(b,a\right)=\sum_{s\in S}b\left(s\right)Q\left(s,a\right)$$ (7)

We have the belief state *b*(*s*) from the Bayesian inference. The state-action value function, Q(s,a), is formally defined as the sum of the expected reward after taking an action *a* in a state *s* [16]:

$$Q\left(s,a\right)=E[r_{t+1}+\max_{a^{\prime }}Q\left(s_{t+1},a^{\prime }|s_{t}=s,a_{t}=a\right)]$$ (8)

where *t* is the time step within one episode. The state-action value function Q(S,A) could be derived using any standard solution for MDP (Markov Decision Process) [16]. Due to the simplicity of our task, Q(S,A) is relatively straightforward to calculate. As mentioned, the underlying state (i.e. the target cell) remained the same within 25 steps (*t* = 1: 25) of each episode. Therefore *s*_*t*+1_ = *s*_*t*_ = *s* (Eq 8). Recall, the state means where the target cell is, so knowing the state means knowing where the target cell is. Therefore after the action at time step *t* (*a*_*t*_ = *a*), the best action is clicking the target cell (denoted as *a*^*s*^) as it gives the highest reward. Therefore, Eq 8 could be expanded as:

$$Q\left(s,a\right)=E[r_{t+1}+Q\left(s,a_{t+1}=a^{s}\right)+Q\left(s,a_{t+2}=a^{s}\right)+\ldots Q\left(s,a_{T}=a^{s}\right)]$$ (9)

where *T* indicates the last time step of the episode, *r*_*t*+1_ is the reward of taking action *a* at state *s* at time step *t*. The term after *r*_*t*+1_ is identical for all the actions at time step *t*, Eq 8 can therefore be further simplified as:

$$Q\left(s,a\right)\propto r_{t+1}\left(s_{t}=s,a_{t}=a\right)$$

As mentioned, one of the constraints was that participants were uncertain about what score they would get given a state and an action, and we modelled this with a likelihood uncertainty parameter Γ. Therefore,

$$r_{t+1}\left(s_{t}=s,a_{t}=a\right)=f\left(s,a\right)=truescore\left(s,a\right)+N\left(0,\Gamma \right)$$ (10)

where *truescore*(*s*, *a*) is calculated based on Eq 2. In summary, we now have an action value of each action given the belief state b based on:

$$Q\left(b,a\right)=\sum_{s\in S}b\left(s\right)Q\left(s,a\right)\to Q\left(b,a\right)=\sum_{s\in S}b\left(s\right)r\left(s,a\right)$$ (11)

The original QMDP algorithm [69] then selects the action that yields the highest value on the belief state. One known disadvantage of the QMDP method is that it lacks exploration. To remedy this shortcoming [71], the action selection in our model was based on the value of each action relative to the value of all the actions (i.e., soft-max action selection). The probability of choosing an action a was calculated as:

$$p\left[a\left(i,j\right)\right]=\frac{e^{\tau \times Q[b,a(i,j)]}}{\sum_{u=1,v=1}^{21,21}e^{\tau \times Q[b,a(u,v)]}}$$ (12)

With *τ* = 0, action selection is totally random. As *τ* increased, the action selection becomes more dependent on the value function Q(B,A). Therefore, the exploration was inversely proportional to the uncertainty in the value function. It has been shown that a temperature schedule that begins with a high level of exploration but then leads to exploration gradually decreasing as learning progresses generally leads to higher expected reward [72]. However, there is no consensus regarding which decreasing schedule is optimal [72]. In our model, *τ* was set to *τ*_0_/*t*, where *τ*_0_ is a constant and *t* is the number of attempts. The *τ*_0_ (between 1 to 15) that generated the highest mean points across 25 attempts was chosen for each Γ (Fig 7). The model’s performance reported in the main results is based on an average over 100 runs.

### Model for the reaching task

Next, a model for the reaching task is introduced by adding motor noise to the model for the decision-making task.

In the decision-making task model, the action space is A=a(i,j), where *i* ∈ [1: 21], *j* ∈ [1: 21]), *a*(*i*, *j*) represents the event of clicking the cell [*i*, *j*] in the grid. That is, each action has two properties *i* and *j*, which represent the cell location in the decision-making task. Given that the participants were able to click the exact cells they wanted during the task, there was no noise/uncertainty between the planned actions and executed actions. In the reaching task, these two properties of an action were the direction and curvature of the movement. Due to motor noise, and a lack of informative visual feedback, there was uncertainty between the planned and executed action.

The model for the reaching task was identical to the decision-making model, except that motor noise was now added (Execution, Fig 6). Recall that, for each participant, we calculated the standard deviations of the direction errors and the curvature errors during the noise measurement task. We used these two standard deviations, *σ*_*dir*_ and *σ*_*cur*_, as the measure of their direction motor noise and curvature motor noise respectively. The motor noise was added to the planned action as follows. The planned action with direction *i* and curvature *j*, *a*[*i*, *j*] became *a*[*i*′, *j*′] due to the motor noise [*σ*_*dir*_, *σ*_*cur*_]:

$$a\left(i^{\prime },j^{\prime }\right)=a\left[i+N\left(0,\sigma_{dir}\right),j+N\left(0,\sigma_{cur}\right)\right]$$ (13)

The motor noise also affects the belief-action value function estimate. Specifically, for the reaching task, the score received after taking action *a* at state *s* (i.e. *r*_*t*+1_|*s*_*t*_ = *s*, *a*_*t*_ = *a*) is also determined by motor noise. That is, even if the target trajectory is known, the reward of executing the planed action is affected by motor noise. Therefore Eq 10 is modified as Eq 14 below.

$$r_{t+1}\left(s_{t}=s,a_{t}=a\right)=f\left(s,a\right)=truescore\left(s,a_{noise}\right)+N\left(0,\Gamma \right)$$ (14)

where *a*_*noise*_ is the action *a* contaminated by motor noise as in Eq 13.

## Supporting information

S1 Fig Learning curve predictions for different target cells (the DM task). The model’s predictions of the learning curves (black) for all the 24 targets used in the experiment, against participant performance (red). Each panel is for a specific target, indicated by blue asterisk plotted against the rectangle in the bottom right of each panel. Red error bars represent 95% CI across 20 participants.
(TIF)

S2 Fig Learning curve predictions for different target trajectories (the MO task). The model’s predictions of the learning curves (black) for all the 24 targets used in the experiment, against participant performance (green). Each panel is for a specific target trajectory, indicated by blue trajectory plotted against the rectangle in the bottom right of each panel. Green error bars represent 95% CI across 20 participants.
(TIF)

S3 Fig Representative participant learning curves for each target with model prediction (average over 100 runs). One participant’s learning curves for all 24 targets in both the DM (red) and the MO task (green), against model predictions (black; average over 100 runs). Each panel represents a specific target.
(TIF)

S4 Fig Representative participant learning curves for each target with model prediction (one single run). One participant’s learning curves for all 24 targets in both the DM (red) and the MO task (green), against model predictions (black; one single run). Each panel represents a specific target.
(TIF)

S1 Table Comparison of the error reduction (Experiment 1). Two-way repeated measures ANOVA results on the three parameters (a,b and c in *y* = *ae*^−*bx*^ + *c*).
(TIF)

S2 Table Comparison of the error reduction (Experiment 2). Two-way repeated measures ANOVA results on the three parameters (a,b and c in *y* = *ae*^−*bx*^ + *c*).
(TIF)

S1 Text Likelihood implementation details. (DOCX)

## References

[1] Lackner JR, Dizio P. Rapid adaptation to Coriolis force perturbations of arm trajectory. Journal of neurophysiology. 1994;72(1):299–313. 7965013 10.1152/jn.1994.72.1.299

[2] Shadmehr R, Mussa-Ivaldi F. Adaptive representation of dynamics during learning of a motor task. Journal of Neuroscience. 1994;14:3208–3224. 8182467 10.1523/JNEUROSCI.14-05-03208.1994 PMC6577492

[3] Martin Ta, Keating JG, Goodkin HP, Bastian AJ, Thach WT. Throwing while looking through prisms. I. Focal olivocerebellar lesions impair adaptation. Brain. 1996;119:1183–1198. 10.1093/brain/119.4.1183 8813282

[4] Miall RC, Jenkinson N, Kulkarni K. Adaptation to rotated visual feedback: A re-examination of motor interference. Experimental Brain Research. 2004;154(2):201–210. 10.1007/s00221-003-1630-2 14608451

[5] Tseng Yw, Diedrichsen J, Krakauer JW, Shadmehr R, Bastian AJ. Sensory prediction errors drive cerebellum-dependent adaptation of reaching. Journal of neurophysiology. 2007;98(1):54–62. 10.1152/jn.00266.2007 17507504

[6] Rabe K, Livne O, Gizewski ER, Aurich V, Beck A, Timmann D, et al Adaptation to visuomotor rotation and force field perturbation is correlated to different brain areas in patients with cerebellar degeneration. Journal of neurophysiology. 2009;101(4):1961–1971. 10.1152/jn.91069.2008 19176608

[7] Diedrichsen J, White O, Newman D, Lally N. Use-Dependent and Error-Based Learning of Motor Behaviors. Journal of Neuroscience. 2010;30(15):5159–5166. 10.1523/JNEUROSCI.5406-09.2010 20392938 PMC6632748

[8] Huang VS, Haith A, Mazzoni P, Krakauer JW. Rethinking Motor Learning and Savings in Adaptation Paradigms: Model-Free Memory for Successful Actions Combines with Internal Models. Neuron. 2011;70(4):787–801. 10.1016/j.neuron.2011.04.012 21609832 PMC3134523

[9] Haith A, Krakauer JW. Theoretical models of motor control and motor learning The Routledge Handbook of Motor Control and Motor Learning. 2013; p. 7–28.

[10] Taylor JA, Ivry RB. Cerebellar and Prefrontal Cortex Contributions to Adaptation, Strategies, and Reinforcement Learning. Progress in Brain Research. 2014;210:217–253. 10.1016/B978-0-444-63356-9.00009-1 24916295 PMC4118688

[11] Izawa J, Shadmehr R. Learning from sensory and reward prediction errors during motor adaptation. PLoS Computational Biology. 2011;7(3). 10.1371/journal.pcbi.1002012 21423711 PMC3053313

[12] Shmuelof L, Huang VS, Haith A, Delnicki RJ, Mazzoni P, Krakauer JW. Overcoming Motor “Forgetting” Through Reinforcement Of Learned Actions. Journal of Neuroscience. 2012;32(42):14617–14621. 10.1523/JNEUROSCI.2184-12.2012 23077047 PMC3525880

[13] Dam G, Kording K, Wei K. Credit Assignment during Movement Reinforcement Learning. PLoS ONE. 2013;8(2). 10.1371/journal.pone.0055352 PMC3568147 23408972

[14] Wu HG, Miyamoto YR, Gonzales Castro LN, Ölveczky BP, Smith MA. Temporal structure of motor vriability is dynamically regulated and predicts motor learning ability. Nature Neuroscience. 2014;17(2):312–321. 10.1038/nn.3616 24413700 PMC4442489

[15] Therrien AS, Wolpert DM, Bastian AJ. Effective Reinforcement learning following cerebellar damage requires a balance between exploration and motor noise. Brain. 2016;139(1):101–114. 10.1093/brain/awv329 26626368 PMC4949390

[16] Sutton RS, Barto AG. Reinforcement Learning: An Introduction. IEEE Transactions on Neural Networks. 1998;9(5):1054–1054. 10.1109/TNN.1998.712192

[17] Wu SW, Delgado MR, Maloney LT. Motor Decision-Making In: Brain Mapping: An Encyclopedic Reference. vol. 3 Elsevier Inc; 2015 p. 417–427.

[18] Kahneman D, Tversky A. Prospect theory: An analysis of decision under risk. Econometrica: Journal of the Econometric Society. 1979; p. 263–291. 10.2307/1914185

[19] Trommershäuser J, Maloney LT, Landy MS. Statistical decision theory and trade-offs in the control of motor response. Spatial vision. 2003;16(3-4):255–275. 10.1163/156856803322467527 12858951

[20] Trommershäuser J, Maloney LT, Landy MS. Decision making, movement planning and statistical decision theory. Trends in Cognitive Sciences. 2008;12(8):291–297. 10.1016/j.tics.2008.04.010 18614390 PMC2678412

[21] Wu SW, Delgado MR, Maloney LT. Economic decision-making compared with an equivalent motor task. Proceedings of the National Academy of Sciences of the United States of America. 2009;106(15):6088–93. 10.1073/pnas.0900102106 19332799 PMC2669401

[22] Wolpert DM, Landy MS. Motor control is decision-making. Current Opinion in Neurobiology. 2012;22(6):996–1003. 10.1016/j.conb.2012.05.003 22647641 PMC3434279

[23] Galea JM, Mallia E, Rothwell J, Diedrichsen J. The dissociable effects of punishment and reward on motor learning. Nature Neuroscience. 2015;18(4):597–602. 10.1038/nn.3956 25706473

[24] van Beers RJ. Motor Learning Is Optimally Tuned to the Properties of Motor Noise. Neuron. 2009;63(3):406–417. 10.1016/j.neuron.2009.06.025 19679079

[25] Kaelbling L, Littman ML, Cassandra A. Planning and Acting in Partially Observable Stochastic Domains. Artificial Intelligence. 1998;101(1-2):99–134. 10.1016/S0004-3702(98)00023-X

[26] Butko NJ, Movellan JR. I-POMDP: An infomax model of eye movement. In: 2008 IEEE 7th International Conference on Development and Learning, ICDL; 2008. p. 139–144.

[27] Rao RPN. Decision making under uncertainty: a neural model based on partially observable markov decision processes. Frontiers in computational neuroscience. 2010;4(11):146 10.3389/fncom.2010.00146 21152255 PMC2998859

[28] Chen X, Lewis RL, Myers C, Houpt J, Howes A. Discovering Computationally Rational Eye Movements in the Distractor Ratio Task In: Reinforcement Learning and Decision Making. Princeton; 2013 p. 106–110.

[29] Chen X, Bailly G, Brumby DP, Oulasvirta A, Howes A. The Emergence of Interactive Behavior: A Model of Rational Menu Search. Proceedings of the ACM CHI’15 Conference on Human Factors in Computing Systems. 2015;1:4217–4226.

[30] Chen X, Starke S, Baber C, Howes A. A Cognitive Model of How People Make Decisions Through Interaction with Visual Displays. In: Proceedings of the ACM CHI’17 Conference on Human Factors in Computing Systems; 2017.

[31] Lewis RL, Howes A, Singh S. Computational rationality: linking mechanism and behavior through bounded utility maximization. Topics in Cognitive Science. 2014;6(2):279–311. 10.1111/tops.12086 24648415

[32] Howes A, Lewis RL, Vera A. Rational adaptation under task and processing constraints: implications for testing theories of cognition and action. Psychological review. 2009;116(4):717–751. 10.1037/a0017187 19839682

[33] Dukas R, Real LA. Effects of recent experience on foraging decisions by Bumble Bees. Oecologia. 1993;94(2):244–246. 10.1007/BF00341323 28314038

[34] Marshall AT, Kirkpatrick K. Relative gains, losses, and reference points in probabilistic choice in rats. PLoS ONE. 2015;10(2). 10.1371/journal.pone.0117697 PMC4319772 25658448

[35] Huys QJM, Eshel N, O’Nions E, Sheridan L, Dayan P, Roiser JP. Bonsai trees in your head: How the pavlovian system sculpts goal-directed choices by pruning decision trees. PLoS Computational Biology. 2012;8(3). 10.1371/journal.pcbi.1002410 22412360 PMC3297555

[36] Montague PR, Dayan P, Person C, Sejnowski TJ. Bee foraging in uncertain environments using predictive hebbian learning. Nature. 1995;377(6551):725–728. 10.1038/377725a0 7477260

[37] Seymour B, Maruyama M, De Martino B. When is a loss a loss? Excitatory and inhibitory processes in loss-related decision-making. Current Opinion in Behavioral Sciences. 2015;5:122–127. 10.1016/j.cobeha.2015.09.003

[38] Koszegi B, Rabin M. Reference-dependent risk attitudes. American Economic Review. 2007;97(4):1047–1073. 10.1257/aer.97.4.1047

[39] Daw ND, Courville AC, Tourtezky DS. Representation and timing in theories of the dopamine system. Neural computation. 2006;18(7):1637–77. 10.1162/neco.2006.18.7.1637 16764517

[40] Dayan P, Daw ND. Decision theory, reinforcement learning, and the brain. Cognitive, affective & behavioral neuroscience. 2008;8(4):429–453. 10.3758/CABN.8.4.429 19033240

[41] Frazier PI, Yu AJ. Sequential hypothesis testing under stochastic deadlines. Advances in Neural Information Processing Systems. 2008; p. 1–8.

[42] Tseng FY, Chao CJ, Feng WY, Hwang SL. Assessment of human color discrimination based on illuminant color, ambient illumination and screen background color for visual display terminal workers. Industrial health. 2010;48(4):438–46. 10.2486/indhealth.MS1009 20720335

[43] Johnson KO, Phillips JR. Tactile spatial resolution. I. two-point discrimination, gap detection, grating resolution, and letter recognition. Journal of neurophysiology. 1981;46(6):1177–1192. 7320742 10.1152/jn.1981.46.6.1177

[44] Schmidt Ra, Bjork Ra. New Conceptualizations of Practice: Common Principles in Three Paradigms Suggest New Concepts for Training. Psychological Science. 1992;3(4):207–217. 10.1111/j.1467-9280.1992.tb00029.x

[45] Shea JB, Morgan RL. Contextual interference effects on the acquisition, retention, and transfer of a motor skill. Journal of Experimental Psychology: Human Learning & Memory. 1979;5(2):179–187.

[46] He K, Liang Y, Abdollahi F, Fisher Bittmann M, Kording K, Wei K. The Statistical Determinants of the Speed of Motor Learning. PLoS Computational Biology. 2016;12(9). 10.1371/journal.pcbi.1005023 PMC5015831 27606808

[47] Frank MJ, Claus ED. Anatomy of a decision: striato-orbitofrontal interactions in reinforcement learning, decision making, and reversal. Psychological review. 2006;113(2):300–326. 10.1037/0033-295X.113.2.300 16637763

[48] Beiser DG, Hua SE, Houk J. Network models of the basal ganglia. Current opinion in neurobiology. 1997;7(2):185–190. 10.1016/S0959-4388(97)80006-2 9142759

[49] Gurney K, Prescott TJ, Redgrave P. A computational model of action selection in the basal ganglia. I. A new functional anatomy. Biological cybernetics. 2001;84(6):401–410. 10.1007/PL00007984 11417052

[50] Frank MJ, Seeberger LC, O’Reilly RC. By carrot or by stick: cognitive reinforcement learning in parkinsonism. Science. 2004;306:1940–1943. 10.1126/science.1102941 15528409

[51] Mink JW. The basal ganglia: Focused selection and inhibition of competing motor programs. Progress in Neurobiology. 1996;50(4):381–425. 10.1016/S0301-0082(96)00042-1 9004351

[52] O’Reilly RC, Frank MJ. Making working memory work: a computational model of learning in the prefrontal cortex and basal ganglia. Neural computation. 2006;18(2):283–328. 10.1162/089976606775093909 16378516

[53] Kringelbach ML, Rolls ET. The functional neuroanatomy of the human orbitofrontal cortex: Evidence from neuroimaging and neuropsychology. Progress in Neurobiology. 2004;72(5):341–372. 10.1016/j.pneurobio.2004.03.006 15157726

[54] Schultz W, Tremblay L, Hollerman JR. Reward processing in primate orbitofrontal cortex and basal ganglia. Cerebral cortex (New York, NY: 1991). 2000;10(3):272–84. 10.1093/cercor/10.3.272 10731222

[55] Bostan AC, Strick PL. The cerebellum and basal ganglia are interconnected. Neuropsychology Review. 2010;20(3):261–270. 10.1007/s11065-010-9143-9 20811947 PMC3325093

[56] O’Reilly JX, Beckmann CF, Tomassini V, Ramnani N, Johansen-Berg H. Distinct and overlapping functional zones in the cerebellum defined by resting state functional connectivity. Cerebral Cortex. 2010;20(4):953–965. 10.1093/cercor/bhp157 19684249 PMC2837094

[57] Miall RC, Galea JM. Cerebellar damage limits reinforcement learning. Commentary on Therrien et al: Effective reinforcement learning following cerebellar damage requires a balance between exploration and motor noise. Brain. 2016;139(1):4–7. 26626368 10.1093/brain/awv329 PMC4949390

[58] Caligiore D, Pezzulo G, Baldassarre G, Bostan AC, Strick PL, Doya K, et al Consensus Paper: Towards a Systems-Level View of Cerebellar Function: the Interplay Between Cerebellum, Basal Ganglia, and Cortex. Cerebellum. 2016; p. 1–27. 26873754 10.1007/s12311-016-0763-3 PMC5243918

[59] Savage LJ. The Theory of Statistical Decision. Journal of the American Statistical Association. 1951;46(253):55–67. 10.1080/01621459.1951.10500768

[60] Von Neumann J, Morgenstern O. Theory of Games and Economic Behavior. Princeton University Press 1944; p. 625.

[61] Körding KP, Wolpert DM. Bayesian decision theory in sensorimotor control. Trends in cognitive sciences. 2006;10(7):319–26. 10.1016/j.tics.2006.05.003 16807063

[62] Nagengast AJ, Braun Da, Wolpert DM. Risk-sensitivity and the mean-variance trade-off: decision making in sensorimotor control. Proceedings Biological sciences / The Royal Society. 2011;278(1716):2325–2332. 10.1098/rspb.2010.2518 PMC3119020 21208966

[63] Barberis NC. Thirty Years of Prospect Theory in Economics: A Review and Assessment. Journal of Economic Perspectives. 2013;27:173–196. 10.1257/jep.27.1.173

[64] Abe M, Schambra H, Wassermann EM, Luckenbaugh D, Schweighofer N, Cohen LG. Reward improves long-term retention of a motor memory through induction of offline memory gains. Current Biology. 2011;21(7):557–562. 10.1016/j.cub.2011.02.030 21419628 PMC3075334

[65] Wächter T, Lungu OV, Liu T, Willingham DT, Ashe J. Differential effect of reward and punishment on procedural learning. The Journal of neuroscience. 2009;29(2):436–43. 10.1523/JNEUROSCI.4132-08.2009 19144843 PMC2765863

[66] Jarvstad A, Hahn U, Rushton SK, Warren Pa. Perceptuo-motor, cognitive, and description-based decision-making seem equally good. Proceedings of the National Academy of Sciences of the United States of America. 2013;110(40):16271–6. 10.1073/pnas.1300239110 24048030 PMC3791786

[67] Silver D, Veness J. Monte-Carlo Planning in Large POMDPs. Advances in neural information processing systems (NIPS). 2010; p. 1–9.

[68] Watkins C, Dayan P. Q-Learning. Machine Learning. 1992;8:279–292. 10.1023/A:1022676722315

[69] Littman ML, Cassandra A, Kaelbling L. Learning policies for partially observable environments: Scaling up. In: Proceedings of the Twelfth International Conference on Machine Learning. February 1970. California: Morgan Kaufmann.; 1995. p. 1–59.

[70] Hauskrecht M. Value-function Approximations for Partially Observable Markov Decision Processes. J Artif Int Res. 2000;13(1):33–94.

[71] Apostolikas A, Tzafestas S. Improved Qmdp Policy for Partially Observable Markov Decision Processes in Large Domains: Embedding Exploration. Intelligent Automation and Soft Computing. 2004;10(3):209–220. 10.1080/10798587.2004.10642878

[72] Vermorel J, Mohri M. Multi-armed bandit algorithms and empirical evaluation. In: Lecture Notes in Computer Science (including subseries Lecture Notes in Artificial Intelligence and Lecture Notes in Bioinformatics). vol. 3720 LNAI; 2005. p. 437–448.

