# Supplementary figures and images for: Predicting explorative motor learning using decision-making and motor noise
Source: PLoS Comput Biol. 2017 Apr 24;13(4):e1005503. doi: 10.1371/journal.pcbi.1005503 (PMC5421818; doi:10.1371/journal.pcbi.1005503)

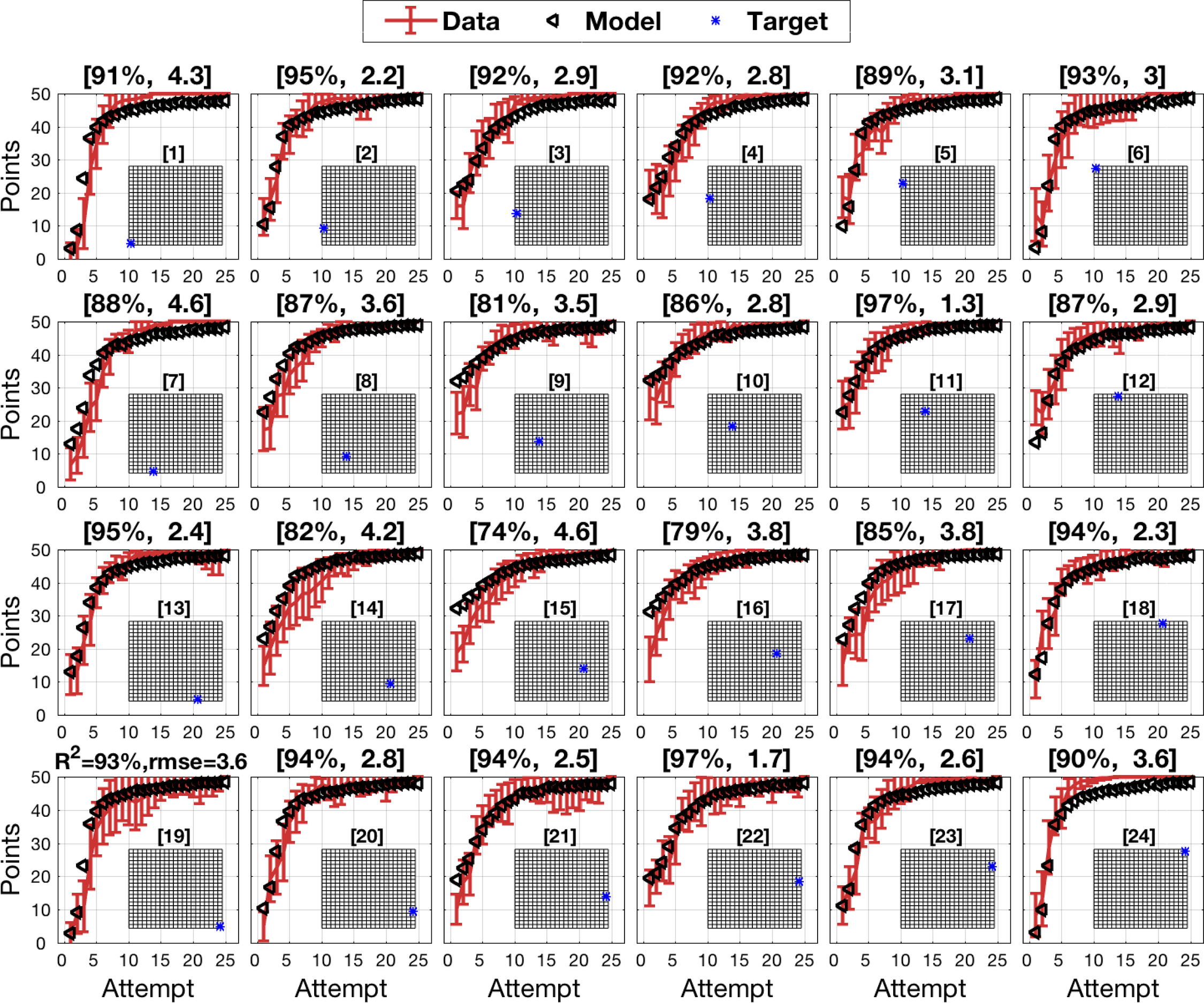

Supplement: S1 Fig — The model’s predictions of the learning curves (black) for all the 24 targets used in the experiment, against participant performance (red). Each panel is for a specific target, indicated by blue asterisk plotted against the rectangle in the bottom right of each panel. Red error bars represent 95% CI across 20 participants. (TIF) [file pcbi.1005503.s001.tif]

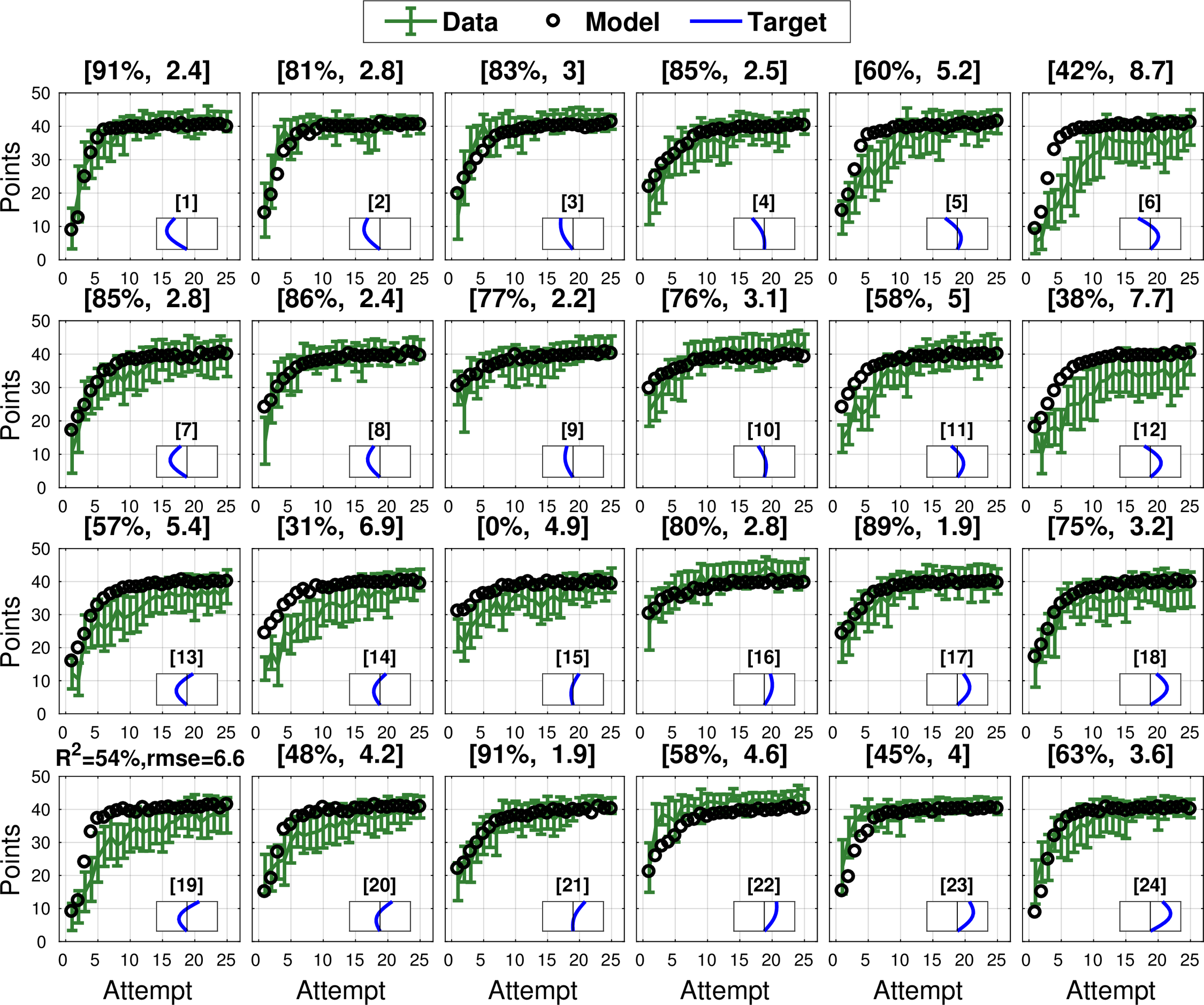

Supplement: S2 Fig — The model’s predictions of the learning curves (black) for all the 24 targets used in the experiment, against participant performance (green). Each panel is for a specific target trajectory, indicated by blue trajectory plotted against the rectangle in the bottom right of each panel. Green error bars represent 95% CI across 20 participants. (TIF) [file pcbi.1005503.s002.tif]

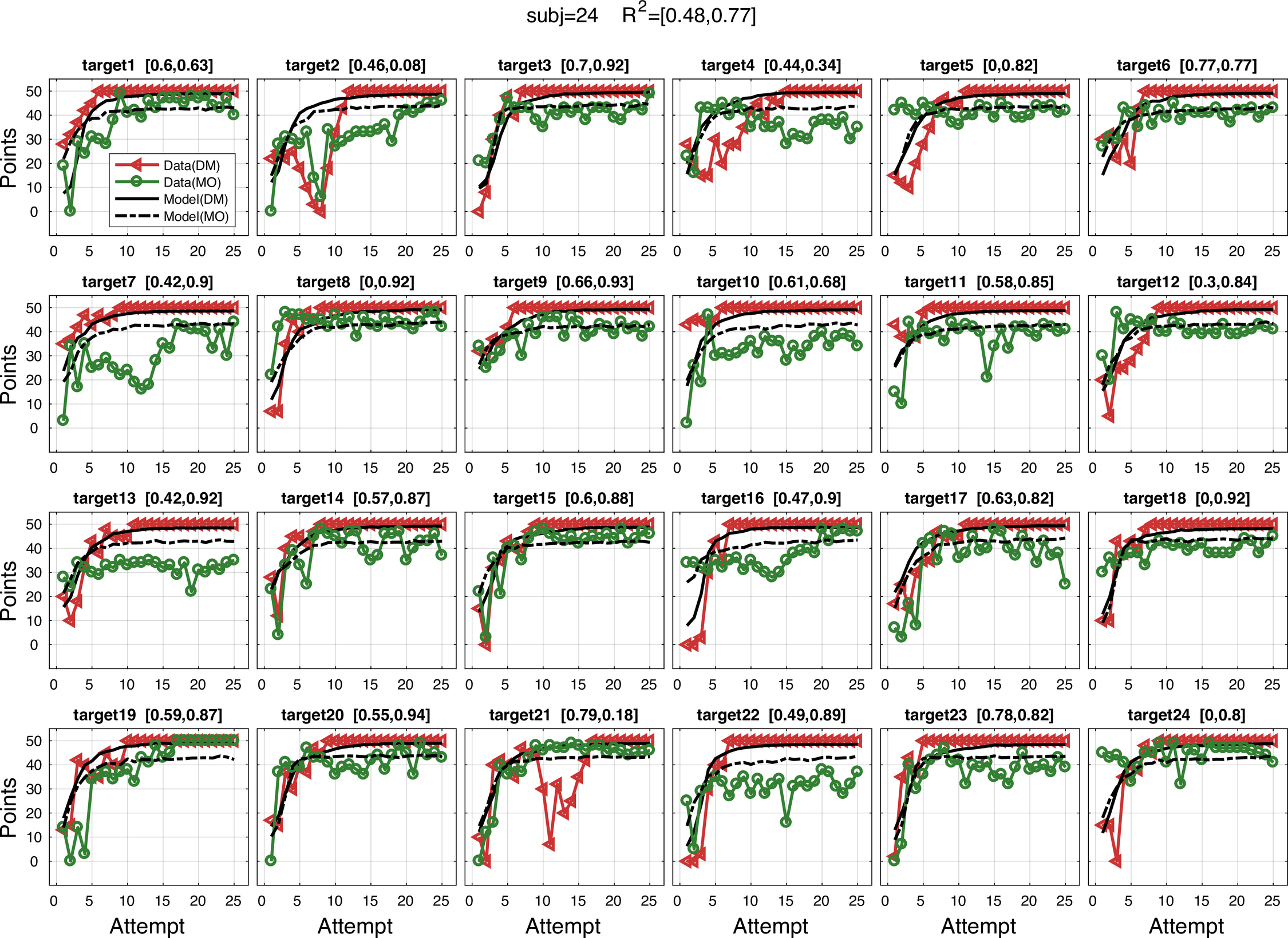

Supplement: S3 Fig — One participant’s learning curves for all 24 targets in both the DM (red) and the MO task (green), against model predictions (black; average over 100 runs). Each panel represents a specific target. (TIF) [file pcbi.1005503.s003.tif]

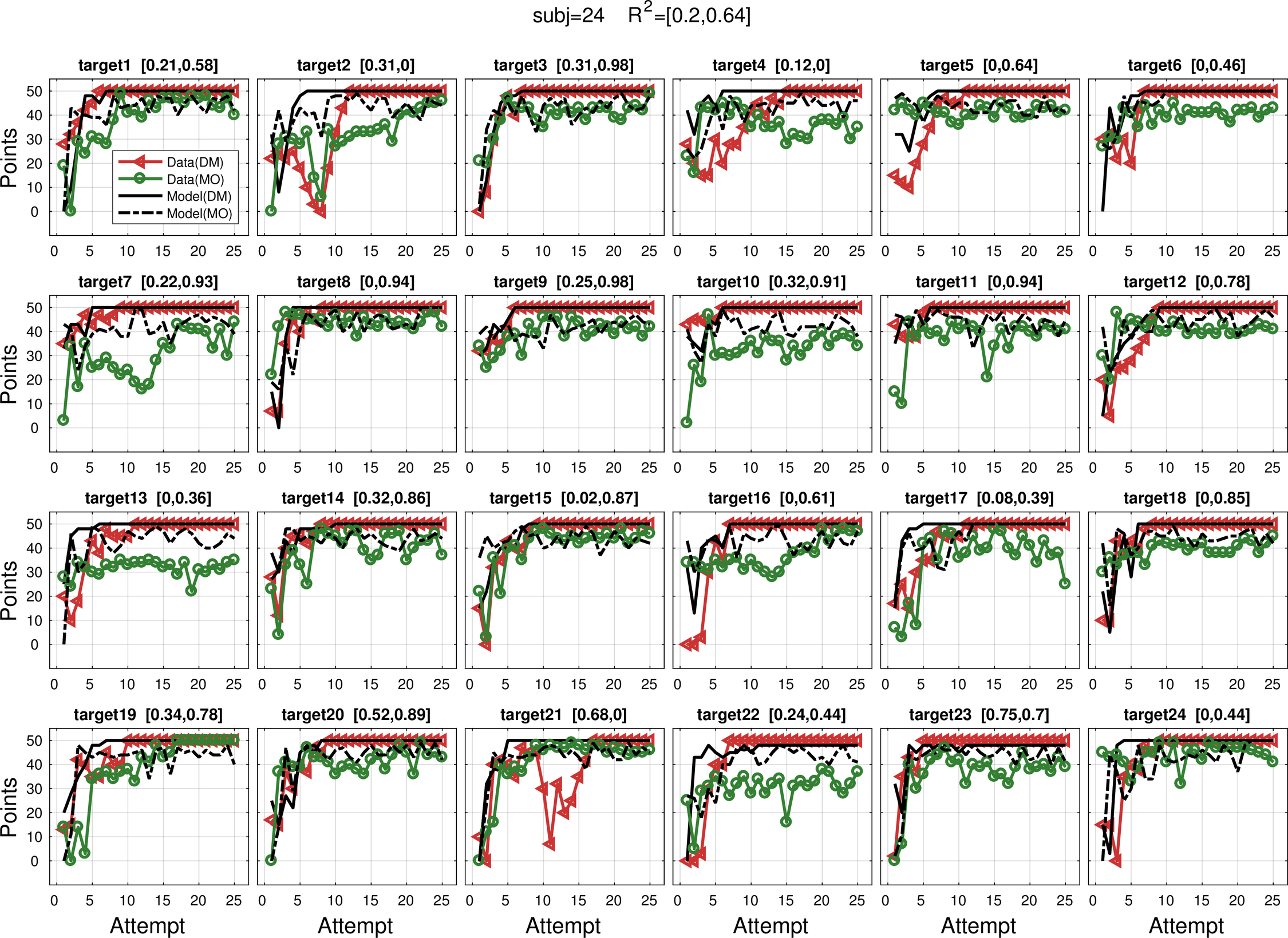

Supplement: S4 Fig — One participant’s learning curves for all 24 targets in both the DM (red) and the MO task (green), against model predictions (black; one single run). Each panel represents a specific target. (TIF) [file pcbi.1005503.s004.tif]

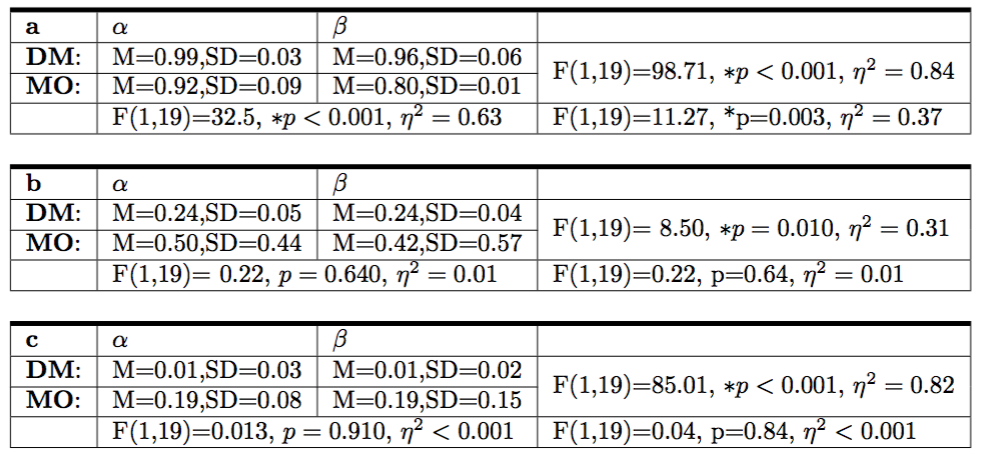

Supplement: S1 Table — Two-way repeated measures ANOVA results on the three parameters (a,b and c in y = ae−bx + c). (TIF) [file pcbi.1005503.s005.tif]

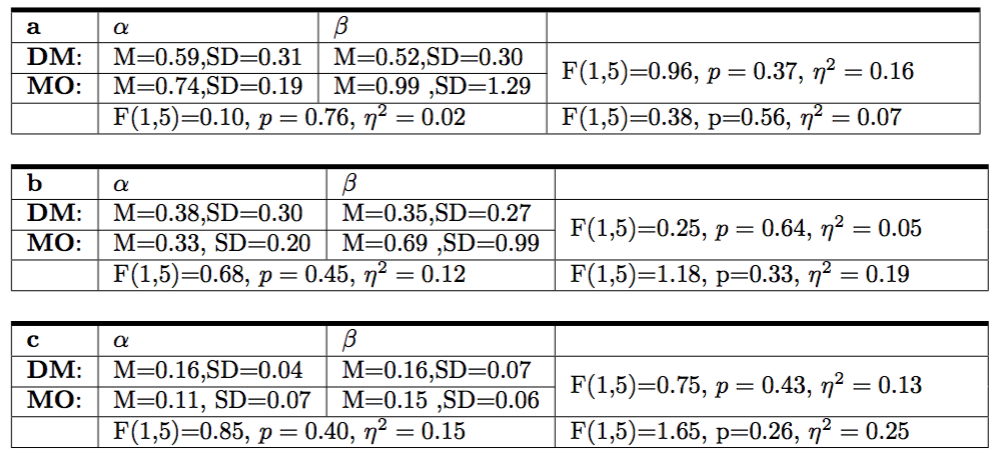

Supplement: S2 Table — Two-way repeated measures ANOVA results on the three parameters (a,b and c in y = ae−bx + c). (TIF) [file pcbi.1005503.s006.tif]
