# Supplementary material for: Predicting explorative motor learning using decision-making and motor noise
Source: PLoS Comput Biol. 2017 Apr 24;13(4):e1005503. doi: 10.1371/journal.pcbi.1005503 (PMC5421818; doi:10.1371/journal.pcbi.1005503)
Supplement: S1 Text — (DOCX) [file pcbi.1005503.s007.docx]

Bayesian update

In our model, the states, actions and observations are all discrete (441 states, 441 actions and 51 observations). The joint distribution of S and O given an action was estimated by sampling.

Specifically: “in the tasks the distribution of the states (S) and actions (A) were both uniform, so the likelihood function p(O|S,A) given the uncertainty (from Gamma and Motor Noise) can be estimated by the following. For each of the state and action pair, we generated 50000 observations (points between 0 to 50) given the Gamma (plus Motor Noise if for the reaching task). Then p(O|S,A) can be directly estimated as the frequency that each of the observations (51 of them) was encountered (within these 50000 samples)”.

Also, besides the human data, all the model code will be made available online (Open Science Framework osf.io/bafms).
